# Potential Gonado-Protective Effect of *Cichorium endivia* and Its Major Phenolic Acids against Methotrexate-Induced Testicular Injury in Mice

**DOI:** 10.3390/biomedicines10081986

**Published:** 2022-08-16

**Authors:** Enas E. Eltamany, Esraa M. Mosalam, Eman T. Mehanna, Basma M. Awad, Sarah M. Mosaad, Maged S. Abdel-Kader, Amany K. Ibrahim, Jihan M. Badr, Marwa S. Goda

**Affiliations:** 1 Department of Pharmacognosy, Faculty of Pharmacy, Suez Canal University, Ismailia 41522, Egypt; 2 Department of Biochemistry, Faculty of Pharmacy, Menoufia University, Shebin El-Koum 32511, Egypt; 3 Department of Biochemistry, Faculty of Pharmacy, Suez Canal University, Ismailia 41522, Egypt; 4 Department of Pharmacognosy, Faculty of Pharmacy, Sinai University, El-Arish 45518, Egypt; 5 Division of Pharmacology and Therapeutics, Department of Continuous Medical Education, General Authority of Healthcare, Ismailia 41522, Egypt; 6 Department of Pharmacognosy, College of Pharmacy, Prince Sattam Bin Abdulaziz University, Al-Kharj 11942, Saudi Arabia; 7 Department of Pharmacognosy, Faculty of Pharmacy, Alexandria University, Alexandria 21215, Egypt

**Keywords:** *C. endivia* extract, phenolic content, antioxidant, methotrexate, testicular injury, inflammation, apoptosis, miR-29a

## Abstract

*Cichorium endivia* L. (Asteraceae) is a wide edible plant that grows in the Mediterranean region. In this study, a phytochemical investigation of *C. endivia* L. ethanolic extract led to the isolation of stigmasterol (**1**), ursolic acid (**2**), *β*-amyrin (**3**), azelaic acid (**4**), vanillic acid (**5**), (6S, 7E)-6-hydroxy-4,7-megastigmadien-3,9-dione (S(+)-dehydrovomifoliol) (**6**), 4-hydroxy phenyl acetic acid (**7**), vomifoliol (**8**), ferulic acid (**9**), protocatechuic acid (**10**), kaempferol (**11**), *p*. coumaric acid (**12**), and luteolin (**13**). In addition, the total phenolic content as well as the in vitro antioxidant activity of *C. endivia* L. extract were estimated. Moreover, we inspected the potential gonado-protective effect of *C. endivia* crude extract, its phenolic fraction, and the isolated coumaric, vanillic, and ferulic acids against methotrexate (MTX)-induced testicular injury in mice. There were seven groups: normal control, MTX control, MTX + *C. endivia* crude extract, MTX + *C. endivia* phenolic fraction, MTX + isolated coumaric acid, MTX + isolated vanillic acid, and MTX + isolated ferulic acid. MTX was given by i.p. injection of a 20 mg/kg single dose. The crude extract and phenolic fraction were given with a dose of 100 mg/kg/day, whereas the compounds were given at a dose of 10 mg/kg/day. A histopathological examination was done. The testosterone level was detected in serum together with the testicular content of malondialdehyde (MDA), catalase (CAT), superoxide dismutase (SOD), interleukin 1β (IL-1β), IL-6, tumor necrosis factor alpha (TNF-α), nuclear factor kappa B (NF-κB), B-cell lymphoma 2 (Bcl-2), Bcl-2 associated x protein (Bax), p53, and miR-29a. *C. endivia* crude extract, the phenolic fraction, and the isolated compounds showed significant elevation in their levels of testosterone, CAT, SOD, Bcl-2 with a significant decrease in their levels of MDA, TNF-α, IL-1β, IL-6, NF-κB, Bax, P53, and miR-29a compared to those of the MTX control group. In conclusion, *C. endivia* mitigated MTX-induced germ cell toxicity via anti-inflammatory, antioxidant, and antiapoptotic effects.

## 1. Introduction

*Cichorium endivia* L. subsp. *pumilum* jacq. (commonly known as chicory) is one of the wild plants that grow in Egypt; it is a wild edible plant distributed in the Mediterranean region and belongs to the family Asteraceae [1,2]. It is also a popular vegetable and nutritionally valuable, with a high content of vitamin C, minerals, and dietary fibers [3]. It is famous among Egyptian farmers and is preferred to be eaten with cheese as a common Egyptian meal [2,4]. Its roots were used traditionally for reducing the symptoms associated with mild digestive disorders including slow digestion, feeling of abdominal fullness, flatulence, and loss of appetite [2], while leaves decoctions were used for poisoning, bacterial infection, rheumatism [5], and diabetes [6].

*C. endivia* L. contains a wide range of medicinally important compounds such as sesquiterpenes and their glycosides [7,8,9,10], flavonoids [11,12,13,14,15], coumarins [16], phenolic acids [8,9,14,17,18,19], and nitrogenous compounds [12,20,21]. 

These valuable contents, besides *C. endivia* L. traditional uses, have encouraged scientists to estimate its biological effects; it has been reported that *C. endivia* L. root’s methanolic extract shows antimicrobial activity especially against Gram-positive bacteria [1]. *C. endivia* L. extract also possesses a powerful in vitro hepatoprotective effect in the HepG2 cell line, where in vivo studies using a *t*-BHP-induced acute liver injury model in mice confirmed these findings [12]. Moreover, it can improve probiotic growth and prevent liver fibrosis caused by (TNBS)-induced intestinal inflammation in rats [22]. The aqueous suspension of *C. endivia* L. leaves powder has shown a protective effect similar to that of the diabetic drug glibenclamide in a streptozotocin-induced diabetic rat model [23]. Moreover, *C. endivia* L. exert a significant induction of apoptosis and the inhibition of proliferation of a human colorectal cancer HCT-8 cell line [24]. The plant root extract has shown a significant cytotoxic effect on breast cancer MCF7cell [25]. In addition, *C. endivia* L. can act as a photosensitizing agent in vivo against a drug-induced benign breast tumor model in rats [26]. Moreover, *C. endivia* L. extract has a high antioxidant potency, which is attributed to the high phenolic content of this plant [6,16,19]. Furthermore, *C. endivia* L. extracts may have a sun-protective ability so that they can be used as sunscreens, as the ethanolic extract of *C. endivia* L. roots can absorb radiation in the UVB spectrum and prohibits a UVB-induced erythema in human’s skin. Moreover, the extract can partly inhibit cell death, IL-6 mRNA expression, and pyrimidine dimer formation in the human keratinocyte cell line subsequent to UVB irradiation [27].

Methotrexate (MTX) is a competitive inhibitor for dihydrofolate reductase (DHFR), and it is used as an anticancer and anti-autoimmune chemotherapeutic agent. MTX has fetal side effects on different organs including the testes [28]. The exact mechanisms by which MTX induces testicular toxicity remain unclear, but several studies have suggested the induction of oxidative stress, inflammation, and apoptosis to be the main culprit in this toxicity [29].

Our present study aims to isolate and identify the chemical structures of the major active ingredients of the plant and to evaluate its antioxidant activity. In addition, the work seeks to investigate the potential gonado-protective effect of *C. endivia* crude extract, its phenolic fraction, the isolated coumaric, vanillic, and ferulic acids against MTX-induced testicular injury in experimental mice through the examination of the antioxidant, anti-inflammatory and anti-apoptotic effect.

## 2. Materials and Methods

### 2.1. Instruments and Chemicals

The ^1^H (400 MHz), ^13^C (100 MHz)-NMR and 2D-NMR spectra were monitored by a JEOL (Freising, Germany) spectrometer while CD_3_OD (Sigma Aldrich^®^, a subsidiary of Merck KGaA, Darmstadt, Germany) and CDCl_3_ (Sigma Aldrich^®^, a subsidiary of Merck KGaA, Darmstadt, Germany) were used as solvents and tetramethylsilane (TMS) was used as an internal standard. Normal-phase silica gel 60 Å, (70–230 mesh, Merck KGaA, Darmstadt, Germany), silica gel 60 Å, (230–400 mesh, Merck KGaA, Darmstadt, Germany) and Sephadex LH-20 (Sigma Aldrich^®^, a subsidiary of Merck KGaA, Darmstadt, Germany) were utilized for column chromatography. Pre-coated aluminum sheets (silica gel 60 F254, 0.25 mm, 20 cm × 20 cm) (Merck, Darmstadt, Germany) were utilized for analytical thin-layer chromatography. SIL G-25 unmodified standard silica-layer glass plates with 2 mm thickness (Macherey-Nagel^®^, Düren, Germany) were utilized for preparative TLC. The visualization of TLC was performed using UV light and an anisaldehyde-sulfuric acid spraying reagent.

### 2.2. Plant Material Collection and Extraction

A *Cichorium endivia* L. subsp. *pumilum* jacq. (Asteraceae) whole plant was gathered from the north of the Sinai Peninsula (Egypt). The plant was authenticated by Prof. Dr. Elsayeda M. Gamal El-Din (Department of Botany, Faculty of Science, Suez Canal University, Ismailia, Egypt). A voucher specimen of *C. endivia* L. (registration number CE-2018) was stored in the herbarium of the Pharmacognosy Department, Faculty of Pharmacy, Suez Canal University. The drying of the collected plant was accomplished in the shade at room temperature for two weeks, then powdered. Three and half kilograms of the whole plant of *C. endivia* L. powder was extracted three times by cold maceration at room temperature with 70% ethanol (35 L, 3 days). The combined extracts were filtered using Whatman’s No.1 filter paper then dried under vacuum at 40 °C to give 360 g of *C. endivia* crude ethanolic extract. 

### 2.3. Determination of Total Phenolic Content “(TPC) Assay”

The quantification of the total phenolic content (TPC) of *C. endivia* L. extract was determined spectrophotometrically via the Folin–Ciocalteu colorimetric method [30,31]. A volume of 3 mL of Folin–Ciocalteau (10%) was mixed with 5 µL (0.05 mL) of *C. endivia* L. crude extract and 0.8 mL of (7.5%) sodium bicarbonate. At room temperature, the reaction solution was incubated for 30 min. Using Milton Roy (Spectronic 1201) spectrophotometer, the mixture’s absorbance was monitored at 765 nm. The TPC result was obtained as mg gallic acid equivalents (GAE)/g extract.

### 2.4. Evaluation of Antioxidant Activity

#### 2.4.1. DPPH Radical-Scavenging Activity

The free radical-scavenging activity of *C. endivia* L. crude extract was estimated with the method reported in [32,33]. Briefly, a 0.004% *w*/*v* methanolic solution of freshly prepared 2,2-diphenyl-1-picrylhydrazyl (DPPH) radical was stored in the dark at 10 °C. A 40 uL of the resultant methanolic solution was added to 3 mL of DPPH solution. The absorbance measurements were monitored immediately via a Milton Roy UV–vis spectrophotometer (Spectronic 1201). The decrease in absorbance at 515 nm was recorded continuously, with the determination of data at 1 min intervals until the stabilization of absorbance (16 min). Without antioxidant control, the absorbances of the DPPH radical and the reference compound, ascorbic acid, were measured. All the recordings were determined in three repetitions then averaged. The inhibition percentage (PI) of the DPPH radical was evaluated according to the formula:

PI = [{(AC − AT)/AC} × 100]

where AC refers to the control’s absorbance at t = 0 min and AT refers to the sample’s absorbance + DPPH at t = 16 min.

The 50% inhibitory concentration (IC_50_) was evaluated from the dose–response curve’s graphic plots using Graphpad Prism software (San Diego, CA, USA).

#### 2.4.2. Ferric Reducing Antioxidant Power (FRAP) Assay

*C. endivia* L. crude extract’s reducing power was evaluated using a method previously reported [34,35]. That method is based on the reduction of ferricyanide corresponding to different concentrations of the extract sample, as the reduction of ferric ion Fe^3+^ to ferrous Fe^2+^ by the extract refers to the antioxidant potential. The extract samples were prepared in 1 mL of methanol then mixed with 2.5 mL of 0.2 M sodium phosphate buffer (at pH 6.6) and 2.5 mL of 1% *w*/*v* potassium ferricyanide [K_3_Fe (CN)_6_] then incubated at 50 °C for 20 min. The reaction mixture was acidified with 2.5 mL of 10% *w*/*v* trichloroacetic acid then centrifuged for 10 min at 1000× *g*. Then, 2.5 mL of deionized water was used for the dilution of 2.5 mL of the supernatant solution then mixed with 0.5 mL of freshly prepared 0.1% *w*/*v* ferric chloride FeCl_3_. The absorbance of the resultant solution was monitored at 700 nm versus a blank using a Milton Roy spectrophotometer (Spectronic 1201). Ascorbic acid was utilized as the reference standard. The reducing capability percentage was calculated according to a formula previously reported in [36].

$$\mathrm{Reducing}\;\mathrm{capability}\;(\%)=100-(\frac{A_{o}-A_{s}}{A_{o}}\times 100)$$

where *Ao* refers to the control solution absorbance and *As* refers to sample absorbance.

#### 2.4.3. Total Antioxidant Capacity (TAC) Assay

The total antioxidant capacity (TAC) of *C. endivia* L. extract was investigated by the phosphomolybdate complex method proposed by [37], which is based on the reduction of Mo (VI) ion to Mo (V) ion by the extract sample producing a green phosphate/Mo (V) complex at acidic pH. A portion of 0.2 mL of methanolic extract solution was mixed with 0.1 mL of the reagent solution (28 mM sodium phosphate, 0.6 M sulfuric acid, and 4 mM ammonium molybdate). This mixture was incubated in a thermal block for 90 min at 95 °C. The sample was cooled then the absorbance was monitored at 695 nm. The TAC result was measured as milligrams of gallic acid equivalents per gram of extract using the standard gallic graph.

### 2.5. Fractionation of C. endivia L. Extract and Isolation of Phytochemical Constituents

To isolate the major phytoconstituents of *C. endivia*, the plant’s crude extract was subjected to a detailed phytochemical investigation applying different preparative chromatographic techniques. About 300 g of *C. endivia* crude extract were first fractionated using VLC with normal-phase silica gel as the stationary phase and using gradient elution with (*n*-hexane/ethyl acetate/methanol) to give 9 fractions (F1–F9) with eluting systems of 100% *n*-hexane (2 L), 75:25 (*n*-hexane/ethyl acetate) (2 L), 50:50 (*n*-hexane/ethyl acetate) (2 L), 25:75 (*n*-hexane/ethyl acetate) (2 L), 100% (EtOAc) (3 L), 75:25 (ethyl acetate/methanol) (3 L), 50:50 (ethyl acetate/methanol) (2 L), 25:75 (ethyl acetate/methanol) and 100% methanol (2 L), respectively. The resulted fractions were dried under reduced pressure. The fractions F3, F4, and F5 showed reasonable yields and promising patterns on TLC. The three fractions were subjected to further chromatographic purifications as follows:

F3: About 6 g was first chromatographed with gradient elution on a normal-phase silica gel column using (*n*-hexane/ethyl acetate/MeOH). The eluate was collected in successive fractions of 20 mL which were monitored by TLC. Finally, the subfractions A1–A9 were given. 

Subfraction A1: About 1.5 g which was eluted by the system of 90:10 (*n*-hexane/ethyl acetate) was rechromatographed with gradient elution on normal-phase flash silica gel column using (*n*-hexane/ethyl acetate/MeOH). The eluate was collected in successive fractions of 5 mL. Subfractions with similar TLC patterns were combined resulting in the isolation of compound **1** (13 mg, white amorphous powder). 

Subfraction A3: About 0.46 g which was eluted by the system of 75:25 (*n*-hexane/ethyl acetate) was rechromatographed on normal-phase silica gel column using gradients of (*n*-hexane/ethyl acetate/MeOH). The eluate was collected in successive fractions of 3 mL. Subfractions with similar TLC patterns were pooled together to give 3 subfractions (A7-S1 to A7-S3). Subfraction A7-S2 (100 mg) was further separated by silica gel column chromatography using isocratic elution with 70:30 (*n*-hexane/ethyl acetate). The eluate was collected in successive fractions of 1 mL. Based on the TLC analysis, similar subfractions were pooled together to obtain two almost-pure substances A7-S2-1 (19 mg) and A7-S1-2 (14 mg). These two substances were finally purified by Sephadex LH-20 using DCM/MeOH (1:1) as the solvent system. The eluate was collected in successive fractions of 0.5 mL to afford two amorphous powders compound **2** (15 mg) and compound **3** (10 mg).

F4: About 7 g was first chromatographed with gradient elution on a normal-phase silica gel column using (n-hexane/ethyl acetate/MeOH). The eluate was collected in successive fractions of 20 mL. Subfractions with similar TLC pattern were combined to give 9 sub-fractions (B1–B9). 

Subfraction B7: About 4 g which was eluted with the system of 65:35 (*n*-hexane/ethyl acetate) was rechromatographed with gradient elution on a normal-phase flash silica gel column using (*n*-hexane/ethyl acetate/MeOH) The eluate was collected in successive fractions of 10 mL, which were analyzed by TLC giving 9 sub-fractions (B7-S1 to B7-S9). Subfraction B7-S3 (200 mg), eluted with the (70:30) *n*-hexane/EtOAc solvent system, was subjected to a Sephadex LH-20 column using (1:1) chloroform/MeOH as a mobile phase. The eluate was collected in successive fractions of 1.5 mL, which were analyzed by TLC to yield compound **4** (12 mg, off-white amorphous powder) and compound **5** (60.4 mg, white amorphous powder). The subfraction (B7-S5) (70 mg), eluted with the (60:40) *n*-hexane/EtOAc solvent system was subjected to Sephadex LH-20 column using 100% methanol as a mobile phase. The eluate was collected in successive fractions of 0.5 mL. Fractions with similar TLC pattern were pooled together to give compound **6** (8 mg, white amorphous powder) and compound **7** (15 mg, yellow to orange needles).

F5: About 5.7 g was chromatographed on normal-phase silica gel with gradient elution using chloroform/MeOH gradients. The eluate was collected in successive fractions of 20 mL. The subfractions (C1–C8) were given based on the TLC analysis.

Subfraction C1 (430 mg), eluted by 100% CHCl_3_, was further fractionated on a silica gel column utilizing isocratic elution by CHCl_3._ The eluate was collected in successive fractions of 3 mL. Subfractions with similar TLC patterns were pooled together to afford 3 subfractions (C1-S1 to C1-S3). Subfraction C1-S3 (80 mg) was further purified using normal-phase PTLC and 5% MeOH in CHCl_3_ as a mobile phase to obtain compound **8** (7 mg, off-white to yellow needles).

Subfraction C2 (960 mg), eluted by the system of 95:5 (CHCl_3_/MeOH), was rechromatographed on a normal-phase flash silica gel column with gradient elution using (CHCl_3_/MeOH) commencing by 100% CHCl_3_ until 15% MeOH in CHCl_3_. The eluate was collected in successive fractions of 5 mL. The subfractions C2-S1 to C2-S5 were obtained (on the basis of the TLC analysis). Subfraction C2-S2 (115 mg) was chromatographed on a Sephadex LH-20 column using 50% MeOH in CHCl_3_. The eluate was collected in successive fractions of 0.5 mL, which were monitored by TLC. Finally, compound **9** (52.3 mg, white amorphous powder) and compound **10** (6 mg, white amorphous powder) were obtained. Subfraction C2-S3 (140 mg) was further purified utilizing a Sephadex LH-20 gel filtration column and 50% MeOH in CHCl_3_ (1:1) as a mobile phase. The eluate was collected in successive fractions of 0.5 mL to obtain compound **11** (8.8 mg, yellow powder) and compound **12** (68.2 mg, white amorphous powder). Subfraction C2-S4 (50 mg) was purified by normal-phase PTLC and a solvent mixture of CHCl_3_/MeOH/HCOOH by a ratio of 85:14:1 as a developer to isolate compound **13** as yellow powder (4.5 mg).

### 2.6. Preparation of the Phenolic Fraction of C. endivia L. Extract

A phenolic portion was prepared by suspending 40 g of *C. endivia* crude extract in 5% aqueous solution of Na_2_CO_3_ followed by solvent portioning with CHCl_3_. The remaining aqueous fraction was neutralized by HCl then fractionated successively between chloroform, ethyl acetate and *n*-butanol. The three extracts were pooled together and concentrated under vacuum [38] to obtain the phenolic fraction of *C. endivia* (27 g).

### 2.7. In Vivo Study

#### 2.7.1. Animals

Male albino mice, weighing 30–35 g, were purchased from The Egyptian Organization for Biological Products and Vaccines (Vacsera, Cairo, Egypt). They were placed in rodent cages supplied with water and food ad libitum and they were subjected to a normal day/night cycle. The mice were left to accommodate for two weeks before the experiments. The study protocol was approved by the ethical committee of the Faculty of Pharmacy at Suez Canal University (approval number: 201803PhDA3). 

#### 2.7.2. Induction of Testicular Injury

Testicular injury was induced in mice by i.p. injection of a single dose of 20 mg/kg of MTX in the fifth day of the experiment duration [29].

#### 2.7.3. Study Design

Seventy male albino mice were divided randomly into seven different groups each with ten mice. The groups were normal control, MTX control, MTX + *Cichorium endivia* crude extract, MTX + *C. endivia* L. phenolic fraction, MTX + coumaric acid, MTX + vanillic acid, and MTX + ferulic acid. The normal control group received normal saline. The crude extract and phenolic fraction were given at a dosage of 100 mg/kg/day, whereas the coumaric, vanillic, and ferulic acids were given at a dosage of 10 mg/kg/day. The extracts in addition to the three isolated acids were dissolved in dimethyl sulfoxide (DMSO)/water (1:2) and the protective duration was continued for 10 days.

#### 2.7.4. Collection of the Samples

Under halothane anesthesia, the mice were sacrificed on the 10th day. The blood samples were gathered in suitable vacutainers to separate the sera by centrifugation. The testicular tissues were removed, washed, and divided into two portions. The first portion was for the determination of the biological parameters and was kept at −80 °C. The second portion was maintained for histopathological examination in 10% formalin.

#### 2.7.5. Histopathological Examination

The testes samples were fixed in paraffin, and then, 3–5 µm sections were stained using hematoxylin and eosin (H&E) and investigated by a pathologist. Images were captured by an Olympus microscope (Japan) supplied with a digital camera. Johnsen’s scoring was used for the grading of the seminiferous tubules based on the presence/absence of the spermatogenic cells and each was given a score from 1–10; a score of 1 represented a poor testicular structure with no seminiferous epithelium, while a score of 10 represented full spermatogenesis [39].

#### 2.7.6. Determination of Biochemical Parameters

##### Determination of Testosterone

The testosterone level was determined in the serum using commercial mouse ELISA kit purchased from MyBioSource (USA, Cat No. MBS777000) according to the supplier’s instructions.

##### Determination of Oxidative Stress Biomarkers

The levels of catalase (CAT), malondialdehyde (MDA), and superoxide dismutase (SOD) were determined in the homogenate of the testicular tissue according to the instructions of the commercial mouse ELISA kit purchased from MyBioSource (San Diego, CA, USA, Cat No. MBS764641, MBS704962, and MBS849290, respectively).

##### Determination of Inflammatory Markers and Apoptosis-Related Proteins

The levels of interleukin 1β (IL-1β), IL-6, Bax, and Bcl-2 were determined by using a mouse ELISA kit purchased from MyBioSource (San Diego, CA, USA, Cat No. MBS701092, MBS590056, MBS763832, and MBS164593, respectively).

##### Determination of Testicular Levels of NF-κB, TNF-α, p53, and miR-29a by Quantitative Real-Time PCR

Total RNA, which includes miRNA, was extracted from testicular tissues by an RNeasy Mini kit (Qiagen, Germany, Cat No. 217004). The expressions of the target genes were established using the GoTaq^®^ 1-Step RT-qPCR System (Promega, Madison, WI, USA). β-actin was used as a reference gene for NF-κB, TNF-α, and p53, while U6B small nuclear RNA (RNU6B) was used for miR-29a (Table 1). The cycling conditions were 15 min at 37 °C for the reverse transcription, 10 min at 95 °C for the inactivation of reverse transcriptase enzyme, followed by 40 cycles of: denaturation at 95 °C for 10 s, annealing for 30 s, and extension at 72 °C for 30 s. The reactions were carried out in a StepOnePlus™ Real-Time PCR thermal cycler (Applied Biosystems, Waltham, MA, USA). ΔΔCt and fold change were calculated, then the results were expressed as mean fold change with respect to the normal control group.

#### 2.7.7. Statistical Analysis

Data were analyzed with the Statistical Package for Social Sciences, SPSS (IBM, Armonk, NY, USA), version 21.0 software. The results were expressed as the mean ± standard deviation (SD). A one-way analysis of variance, ANOVA, then a Bonferroni’s post hoc test were used for the statistical analysis. A *p* < 0.05 was considered significant statistically.

## 3. Results

### 3.1. Total Phenolic Content (TPC)

Phenolic compounds are phytochemicals which are widely dispersed throughout the plant kingdom. They can be classified into flavonoids and nonflavonoids (such as phenolic acids, coumarins, lignans, etc.). The interest in phenolic compounds has recently increased due to their biological activities and promising therapeutic effects [40]. Thus, the determination of the phenolic content in the extract was necessary to evaluate the potential antioxidant capacity of *C. endivia* L. crude extract. The total phenolic content (TPC) of *C. endivia* L. crude extract was 26.41 ± 2.35 mg GAE/g of plant extract, and it was evaluated spectrophotometrically using the Folin–Ciocalteu colorimetric method.

### 3.2. Evaluation of In Vitro Antioxidant Activity of C. endivia L. Extract

**Figure 1:**
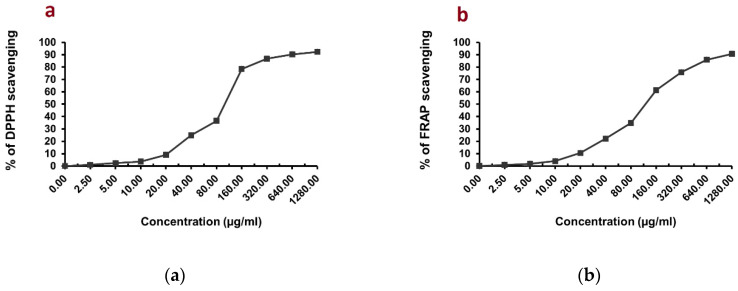
(**a**) The scavenging rate (%) of DPPH and (**b**) the scavenging rate (%) of FRAP by *C. endivia* L. crude extract. All values in the figure are expressed as means (%) and SD of triplicated experiments.

**Figure 2:**
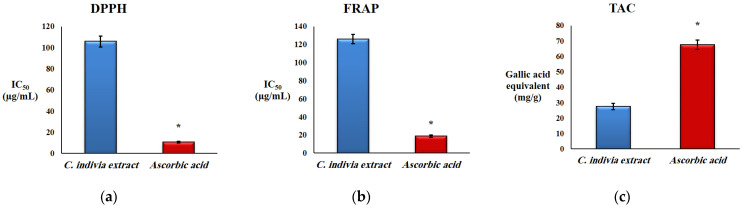
Antioxidant activity of *C. endivia* L. crude extract: (**a**) the IC_50_ value of DPPH radical scavenging assay; (**b**) the IC_50_ value of FRAP scavenging assay; and (**c**) total antioxidant capacity (TAC) assay. Results are expressed as mean ± SD and compared by Student *t* test. * Significantly different compared to *C. endivia* crude extract at *p* < 0.05.

In this study, three tests (DPPH, FRAP, TAC) were performed to evaluate the antioxidant potential of *C. endivia* L. crude extract. Figure 1a,b shows that *C. endivia* L. crude extract had definite scavenging activities with DPPH and FRAP assays displaying a dose-dependent scavenging rate. In Figure 2a, *C. endivia* L. crude extract with an IC_50_ value of 105.66 ± 4.98 µg/mL exhibited a marked activity in the DPPH radical scavenging assay compared to that of the ascorbic acid reference standard with IC_50_ = 10.64 ± 0.82 µg/mL. Figure 2b shows the FRAP assay results and demonstrates the reduction potential of *C. endivia* L. with IC_50_ = 125.71 ± 5.27 µg/mL in comparison to ascorbic acid reference standard with IC_50_ = 18.7 ± 1.26 µg/mL. Figure 2c shows the total antioxidant capacity (TAC) of *C. endivia* L. extract with a remarkable antioxidant potential of 27.43 ± 2.09 mg GAE/g in comparison with ascorbic acid as a reference standard antioxidant with the antioxidant potential of 67.48 ± 3.14 mg GAE/g, and it was evaluated using a phosphomolybdate complex method. 

### 3.3. Chemical Identification of the Isolated Compounds

**Figure 3:**
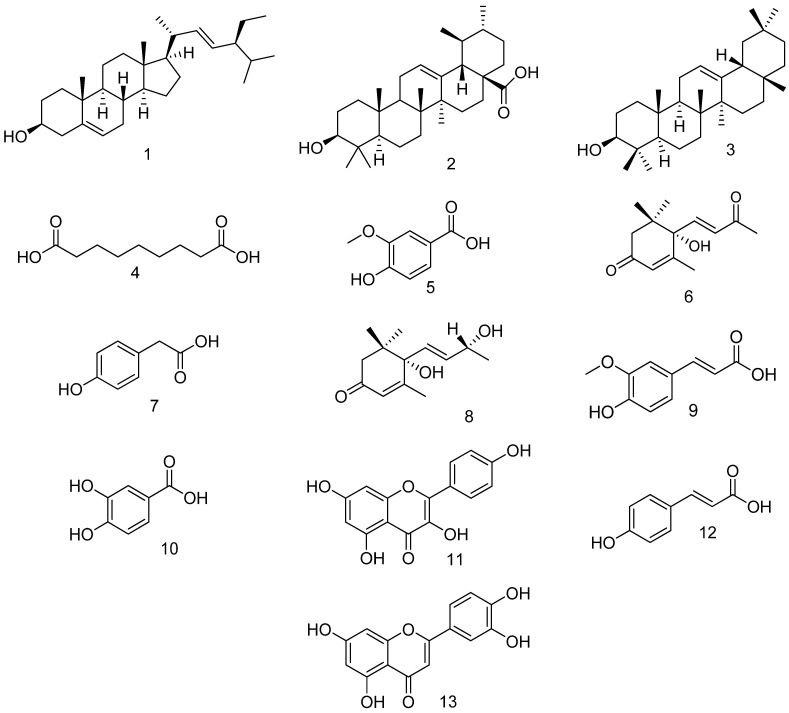
Chemical structure of the isolated compounds.

The phytochemical investigation of *C. endivia* L. yielded thirteen compounds (Figure 3). The chemical identification of the isolated compounds **1**, **4**–**10**, and **12** was accomplished by the comparison of their ^1^H-NMR and ^13^C-NMR spectral data with those previously published in literature. All the NMR spectral data of these compounds were in agreement with the published results. Hence, their chemical structures were assigned to be: stigmasterol (**1**), azelaic acid (**4**), vanillic acid (**5**), (6S, 7E)-6-hydroxy-4,7-megastigmadien-3,9-dione (S(+)-dehydrovomifoliol) (**6**), 4-hydroxy phenyl acetic acid (**7**), vomifoliol (**8**), ferulic acid (**9**), protocatechuic acid (**10**), kaempferol (**11**), *p*-coumaric acid (**12**), and luteolin (**13**). The other compounds **2**, **3**, **11** and **13** were identified as ursolic acid, *β*-amyrin, kaempferol, and luteolin, respectively, by cochromatography with authentic samples. 

NMR spectral data of the isolated compounds:

Stigmasterol (**1**), (Figures S1 and S2):

^1^H NMR (400 MHz, CDCl_3_) *δ*, 5.37 (d, *J* = 4.9 Hz, 1H), 5.17 (dd, *J* = 8.6, 8.6Hz, 1H), 5.04 (dd, *J* = 8.6, 8.6 Hz, 1H), 3.54 (m, 1H), 2.29 (m, 2H), 1.05 (s, 3H), 1.03 (s, 3H), 0.86 (d, *J* = 6.1 Hz, 3H), 0.82 (s, 1H), 0.81 (s, 3H), 0.72 (s, 3H).

^13^C NMR (100 MHz, CDCl_3_) *δ* 140.77 (C-5), 138.24 (C-22), 129.19 (C-23), 121.50 (C-6), 71.32 (C-3), 56.79 (C-14), 55.88 (C-17), 51.17 (C-24), 50.09 (C-9), 42.13 (C-4), 41.85 (C-13), 40.41 (C-20), 39.61 (C-12), 37.18 (C-1), 36.44 (C-10),32.33 (C-25), 31.82 (C-7), 31.80 (C-8), 31.17 (C-2), 28.83 (C-16), 25.31 (C-28), 24.26 (C-15), 21.08 (C-27), 20.98 (C-21), 20.94 (C-11), 19.24 (C-19), 18.84 (C-26), 12.09 (C-18), 11.73 (C-29) [41].

Ursolic acid (**2**): identified by cochromatography (R_f_ = 0.71; mobile phase: 3% methanol in CHCl_3_) [42].

*β*-amyrin (**3**): Identified by cochromatography (R_f_ = 0.39; mobile phase: 25% EtOAc in hexane) [42].

Azelaic acid (**4**), (Figures S3 and S4):

^1^H NMR (400 MHz, CD_3_OD) *δ* 2.30 (t, *J* = 7.4 Hz, 4H), 1.62 (p, *J* = 7.0 Hz, 4H), 1.30–1.41 (m, 6H), which is first isolated from *C. endivia* L.

^13^C NMR (100 MHz, CD_3_OD) *δ* 176.44 (C-1, 9), 33.60 (C-2, 8), 28.66 (C-3, 7), 24.65 (C-4, 5,6) [43].

Vanillic acid (**5**), (Figures S5–S7):

^1^H NMR (400 MHz, CD_3_OD) *δ* 7.58 (dd, *J* = 6.8, 2.0 Hz, 1H), 7.57 (d, *J* = 2.0 Hz, 1H), 6.86 (d, *J* = 6.8 Hz, 1H), 3.89 (s, 3H).

^13^C NMR (100 MHz, CD_3_OD) *δ* 168.73 (C=O), 151.22 (C-3), 147.24 (C-4), 123.91 (C-1), 121.63 (C-6), 114.46 (C-2), 112.39 (C-5), 55.02 (OCH_3_) [44].

(6*S*, 7*E*)-6-hydroxy-4,7-megastigmadien-3,9-dione (*S* (+)-dehydrovomifoliol) (**6**), (Figures S8–S12):

^1^H NMR (400 MHz, CD_3_OD) *δ* 7.01 (d, *J* = 15.8 Hz, 1H), 6.46 (d, *J* = 15.8 Hz, 1H), 5.96 (t like, 1H), 2.61 (d, *J* = 17.2 Hz, 1Hb), 2.33 (s, 1H), 2.30 (d, *J* = 17.2 Hz, 1Ha), 1.92 (d, *J* = 1.2 Hz, 1H), 1.08 (s, 1H), 1.04 (s, 1H).

^13^C NMR (101 MHz, CD_3_OD) *δ* 199.13 (C-3/C-9), 163.31 (C-5), 147.00 (C-7), 130.32 (C-8), 126.64 (C-4), 78.60 (C-6), 49.13 (C-2), 41.26 (C-1), 29.37 (C-10), 23.32 (C-11), 22.06 (C-12), 17.78 (C-13) [45].

4-Hydroxy phenyl acetic acid (**7**), (Figures S13–S15):

^1^H NMR (400 MHz, CD_3_OD) *δ* 7.11 (d, *J* = 8.5 Hz, 2H), 6.75 (d, *J* = 8.5 Hz, 2H), 3.50 (s, 2H).

^13^C NMR (100 MHz, CD_3_OD) *δ* 175.10 (C=O), 155.94 (C-4), 129.95 (C2, 6), 125.55 (C-1), 114.84 (3, 5), 39.90 (C-7) [16].

Vomifoliol (**8**), (Figures S16–S20):

^1^H NMR (400 MHz, CD_3_OD) *δ* 5.90 (t, *J* = 1.4 Hz, 1H), 5.82–5.81 (m, 1H), 5.81–5.79 (m, 1H), 4.34 (qd, *J* = 6.4, 3.8 Hz, 1H), 2.53 (d, *J* = 16.9 Hz, 1Hb), 2.18 (d, *J* = 16.8 Hz, 1Ha), 1.94 (d, *J* = 1.6 Hz, 3H), 1.26 (d, *J* = 6.5 Hz, 3H), 1.06 (s, 3H), 1.03 (s, 3H).

^13^C NMR (100 MHz, CD_3_OD) *δ* 199.90 (C-3), 166.14 (C-5), 135.52 (C-8), 128.69 (C-7), 125.70 (C-4), 78.57 (C-6), 67.33 (C-9), 49.35 (C-2), 41.05 (C-1), 23.10 (C-12), 22.45 (C-10), 22.09 (C-11), 18.21 (C-13) [46,47], which is first isolated from *C. endivia* L.

Ferulic acid (**9**), (Figures S21–S23):

^1^H NMR (400 MHz, CD_3_OD) *δ* 7.61 (d, *J* = 15.9 Hz, 1H), 7.19 (d, *J* = 2.0 Hz, 1H), 7.08 (dd, *J* = 8.2, 2.0 Hz, 1H), 6.84 (d, *J* = 8.2 Hz, 1H), 6.33 (d, *J* = 15.9 Hz, 1H), 3.91 (s, 3H).

^13^C NMR (100 MHz, CD_3_OD) *δ* 169.68 (C=O), 149.04 (C-4), 147.97 (C-3), 145.55 (C-7), 126.41 (C-1), 122.59 (C-6), 115.09 (C-8), 114.52 (C-5), 110.32 (C-2), 55.08 (OCH_3_) [48].

Protocatechuic acid (**10**), (Figures S24–S28):

^1^H NMR (400 MHz, CD_3_OD) *δ* 7.46 (d, *J* = 2.1 Hz, 1H), 7.44 (dd, *J* = 8.5, 2.1 Hz, 1H), 6.82 (d, *J* = 8.5 Hz, 1H).

^13^C NMR (100 MHz, CD_3_OD) *δ* 169.28 (C-7), 149.95 (C-4), 144.59 (C-3), 122.48 (C-6), 122.23 (C-1), 116.38 (C-5), 114.38 (C-2) [44].

Kaempferol (**11**): identified by cochromatography (R_f_ = 0.26; mobile phase: toluene/ethyl acetate/formic acid 7:3:0.1 [49].

*p*-Coumaric acid (**12**), (Figures S29–S31):

^1^H NMR (400 MHz, CD_3_OD) *δ* 7.62 (d, *J* = 15.8 Hz, 1H), 7.45 (d, *J* = 8.6 Hz, 2H), 6.82 (d, *J* = 8.6 Hz, 2H), 6.29 (d, *J* = 15.8 Hz, 1H).

^13^C NMR (100 MHz, CD_3_OD) *δ* 169.73 (C=O), 159.69 (C-4), 145.35 (C-7), 129.72 (C-2/C-6), 125.86 (C-1), 115.45 (C-8), 114.18 (C-3/C-5) [48].

Luteolin (**13**): identified by cochromatography (R_f_ = 0.32; mobile phase: toluene/ether/acetic acid (6:4:1)) [50].

### 3.4. In Vivo Study

#### 3.4.1. Histopathological Finding

**Figure 4:**
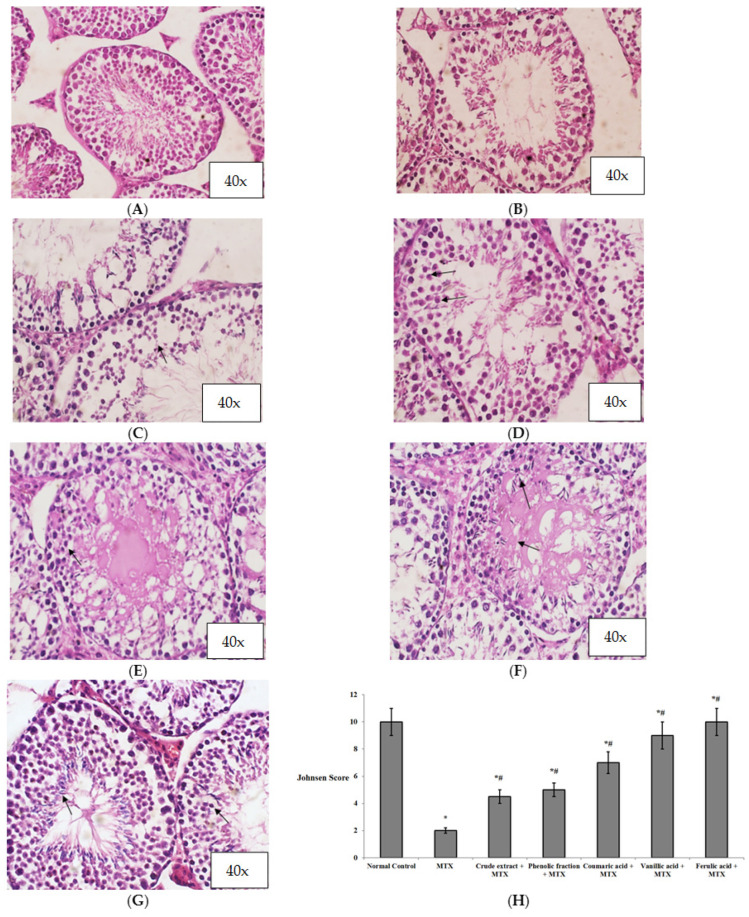
Histopathological examination of the effects of *C. endivia* L. crude extract, phenolic fraction, and its isolated compounds (coumaric, vanillic, and ferulic acids) on the testicular tissue in the experimental mice (40×). (**A**) Normal control group, (**B**) MTX control group, (**C**) *C. endivia* L. crude-extract-treated group, (**D**) *C. endivia* L. phenolic-extract-treated group, (**E**) coumaric acid treated group, (**F**) vanillic acid treated group, (**G**) ferulic acid treated group, and (**H**) bar charts representing Johnsen’s scoring for the testes in the studied groups. Black arrows refer to spermatogenesis. Data are expressed as the mean ± SD. Data were analyzed using one-way ANOVA then Bonferroni’s post hoc test. * Significant against normal control group, # significant against MTX control group. Values were considered significantly different at *p* < 0.05.

Figure 4A shows that the normal control group exhibited full spermatogenesis up to the level of spermatozoa. In contrast, the MTX group revealed some hyalinized tubules, while other tubules showed a significant reduction in spermatogenesis and only a few spermatocytes (Figure 4B), since MTX is widely used to induce testicular injury owing to its toxicity on germ cells. The pretreatment with *C. endivia* L. crude extract showed few spermatocytes (Figure 4C), whereas the phenolic fraction showed many spermatocytes (Figure 4D). The pretreatment with the coumaric acid exhibited reduced germinal cells in about 30% of the tubules with few spermatocytes with hyalinized lumen, and other tubules showed full spermatogenesis as shown in Figure 4E. The vanillic acid pretreated group exhibited full spermatogenesis, up to the level of spermatids, despite the disturbed lining epithelium and the hyalinized tubal lumens (Figure 4F). In the same manner, the ferulic acid pretreated group showed full spermatogenesis up to the level of spermatozoa (Figure 4G).

Figure 4H shows the statistical analysis of Johnsen’s scoring in the studied groups. The MTX group (score 2) showed a significant decrease in the score compared to the normal control group. All of the groups pretreated with the *C. endivia* L. crude extract, phenolic fraction, and isolated acids showed significant increases in Johnsen’s scoring of the testes in comparison with the MTX control group. The best improvement in testicular architecture was revealed by ferulic acid (score 10).

#### 3.4.2. Effect on Biochemical Parameters

**Figure 5:**
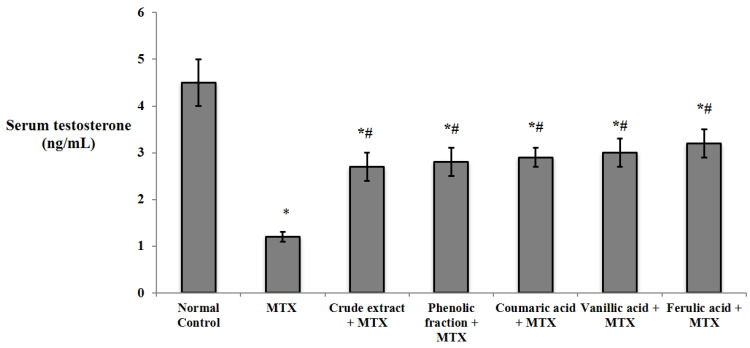
Effects of *C. endivia* L. crude extract, phenolic fraction, and its isolated compounds (coumaric, vanillic, and ferulic acids) on the serum level of testosterone in the experimental mice. Data are expressed as the mean ± SD. Data were analyzed using one-way ANOVA followed by Bonferroni’s post hoc test. * Significant against normal control group, # significant against MTX control group. Values were considered significantly different at *p* < 0.05.

The induction of testicular injury by MTX significantly reduced the serum level of testosterone compared to that of the normal control. The pretreatment with *C. endivia* L. crude extract, phenolic fraction, and the isolated coumaric, vanillic, and ferulic acids showed a significant elevation of the serum level of testosterone relatively to the MTX control group as shown in Figure 5. 

**Figure 6:**
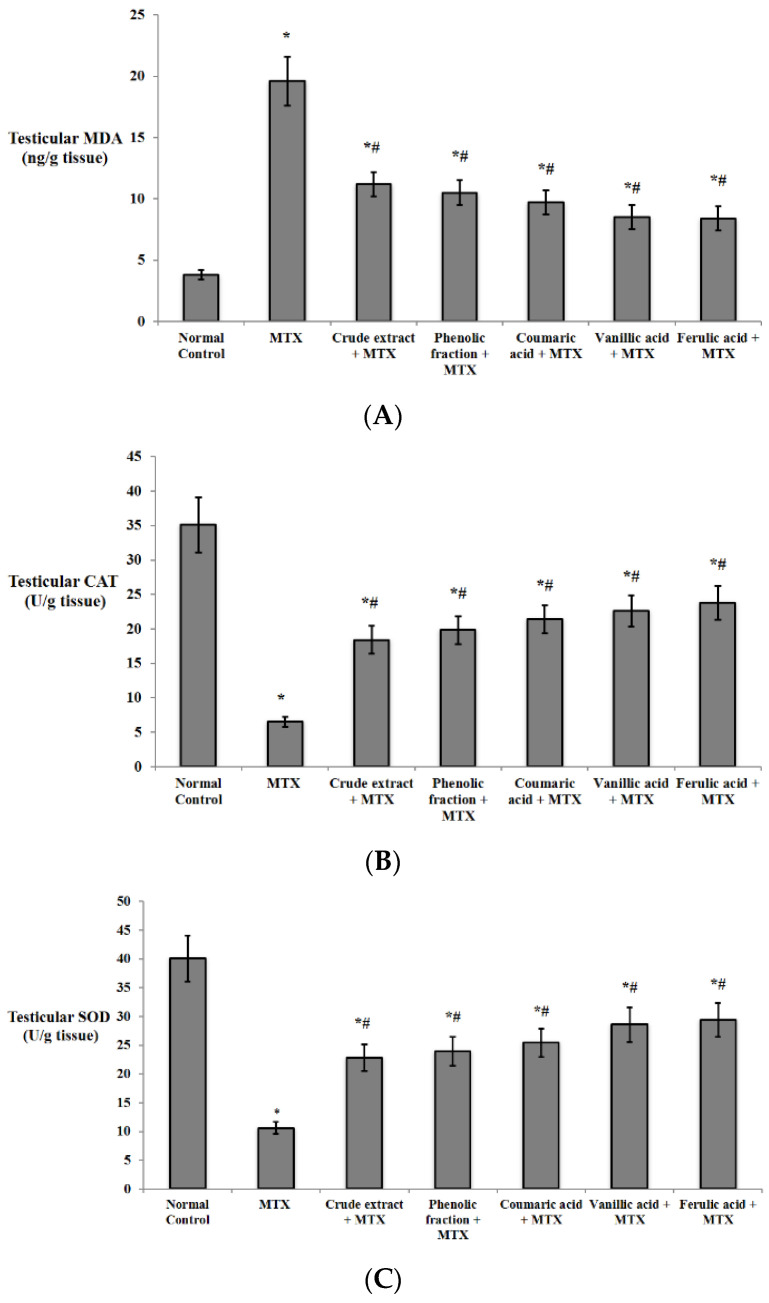
Effects of *C. endivia* L. crude extract, phenolic fraction, and its isolated compounds (coumaric, vanillic, and ferulic acids) on the testicular oxidative stress biomarkers in the experimental mice. (**A**) MDA, (**B**) CAT, and (**C**) SOD. Data are expressed as the mean ± SD. Data were analyzed using one-way ANOVA then Bonferroni’s post hoc test. * Significant against normal control group, # significant against MTX control group. Values were considered different significantly at *p* < 0.05. MDA: malondialdehyde, CAT: catalase, SOD: superoxide dismutase.

Regarding the effect on the oxidative stress biomarkers, MTX exhibited a significant increase in the testicular content of MDA with a significant decrease in the testicular CAT and SOD comparable to those of the normal control group. The pretreatment with *C. endivia* L. crude extract, phenolic fraction, and the three acids demonstrated a significant decline in the concentration of MDA with a significant increase in the concentration of CAT and SOD compared to those of the MTX control group as presented in Figure 6.

**Figure 7:**
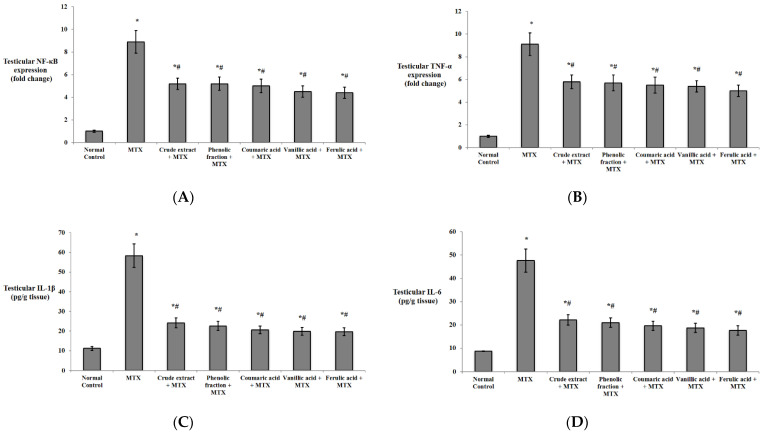
Effects of *C. endivia* L. crude extract, phenolic fraction, and its isolated compounds (coumaric, vanillic, and ferulic acids) on the testicular inflammatory biomarkers in the experimental mice. (**A**) NF-κB expression, (**B**) TNF-α expression, (**C**) IL-1β levels, and (**D**) IL-6 levels. Data are expressed as the mean ± SD. Data were analyzed using one-way ANOVA then Bonferroni’s post hoc test. * Significant against normal control group, # significant against MTX control group. Values were considered different significantly at *p* < 0.05. NF-κB: nuclear factor-kappa B, TNF-α: tumor necrosis factor-alpha, IL-1β: interleukin 1β, IL-6: interleukin 6.

Comparing with the normal group, the testicular injury control group showed a significant upregulation of NF-κB and TNF-α, with a significant increase in the levels of the inflammatory markers IL-1β and IL-6. On the contrary, giving the mice the crude extract, the phenolic fraction, and coumaric, vanillic, and ferulic acids significantly downregulated those biomarkers relative to those of the testicular injury control as shown in Figure 7.

**Figure 8:**
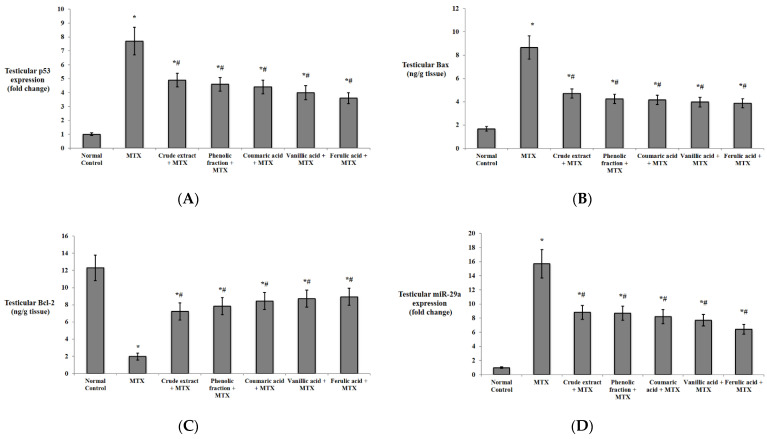
Effects of *C. endivia* L. crude extract, phenolic fraction, and its isolated compounds (coumaric, vanillic, and ferulic acids) on the testicular apoptosis biomarkers in the experimental mice. (**A**) p53 expression, (**B**) Bax levels, (**C**) Bcl-2 levels, and (**D**) miR-29a expression. Data are expressed as the mean ± SD. Data were analyzed using one-way ANOVA followed by Bonferroni’s post hoc test. * Significant against normal control group, # significant against MTX control group. Values were considered different significantly at *p* < 0.05. Bax: Bcl-2-associated X protein, Bcl-2: B-cell lymphoma.

Concerning apoptosis regulatory proteins, the MTX group significantly increased p53 expression and Bax protein levels with a significant decrease in Bcl-2 levels in comparison with the normal group. The pretreatment with the crude extract, the phenolic fraction, and the isolated acids showed a significant decline in p53 expression and Bax levels together with a significant elevation in Bcl-2 levels compared to those of the MTX group. MTX significantly upregulated miR-29a compared to the normal group, whereas the extract, the phenolic fraction, and the isolated acids significantly downregulated miR-29a compared to the MTX control group (Figure 8).

It is worth mentioning that the superior pharmacological activity regarding antioxidant, anti-inflammatory, and antiapoptotic activities was expressed by the ferulic acid pretreated group. Consequently, the testosterone level in this group was significantly elevated with a Johnsen’s scoring of the testes of 10.

## 4. Discussion

*C. endivia* is a cheap edible plant with a high nutritional value [3] used traditionally as a digestive aid [2], as well as for the treatment of chronic diseases such as rheumatism [5] and diabetes [6]. The plant is known to accumulate terpenoids [7,8,9,10], phenolics [8,9,11,12,13,14,15,16,17,18,19], and nitrogenous compounds [12,20,21]. In addition, several reports have shown evidence of *C. endivia*’s diverse bioactivities [1,6,12,16,19,22,23,24,25,26,27]. However, the protective effect of this plant or/and its phytochemicals against chemotherapy-triggered organ toxicities has not been studied yet. Motivated by the above-mentioned considerations, the current study was designed to investigate the protective effect of *C. endivia* crude extract as well as its major chemical constituents against methotrexate-associated gonadal toxicity in male mice Therefore, the crude extract of *C. endivia* was subjected to extensive chromatographic investigation in a pursuit to isolate its major components. As a consequent, thirteen known compounds were isolated. They were the triterpenes: stigmasterol, *β*-amyrin, and ursolic acid; the megastigmane-type norisoprenoid: (6*S*, 7*E*)-6-hydroxy-4,7-megastigmadien-3,9-dione (*S* (+)-dehydrovomifoliol) and vomifoliol; the phenolic acids: ferulic, *p*-coumaric, protocatechuic, vanillic, and 4-hydroxy phenyl acetic acids; the flavonoids: kaempferol and luteolin; and the dicarboxylic acid: azelaic acid. All the isolated compounds were reported previously in the plant [12,13,14,15,16,18,45] except azelaic acid and vomifoliol, which are isolated for the first time from *C. endivia.*

Most of the isolated compounds were phenolics, which reflected that *C. endivia* is a rich source of such compounds. Therefore, the total phenolic content (TPC) of *C. endivia* L. crude extract was quantified spectrophotometrically using the Folin–Ciocalteu (FC) colorimetric assay. This method fundamentally is based on SET for the oxidation of phenol compounds in alkaline medium with a molybdotungstophosphate hetero-polyanion reagent (3H_2_O-P_2_O_5_-13WO_3_-5MoO_3_-10H_2_O), where the molybdenum center in the complex reagent is reduced from Mo (VI) to Mo(V) yielding a blue-colored product [51]. This conventional FC assay is suitable for only the estimation of hydrophilic antioxidants such as polyphenolic compounds since this test is chiefly performed in an aqueous phase that makes it inapplicable for the determination of lipophilic ones [51,52,53].

The obtained results demonstrated that the TPC of *C. endivia* crude extract had a value of 26.41 ± 2.35 mg GAE/g, which is compatible with what was reported by Khalil and Kamel [16]. It is noteworthy to mention that the TPC of *C. endivia* crude extract was greater than that of *C. intybus* leaves (wild chicory): 25.93 ± 0.20 mg/g GAE for the methanolic extract and 21.01 ± 0.47 mg/g GAE for the ethanolic extract. Meanwhile, the TPC of the cultivated variety of *C. intybus* was 19.21 ± 0.60 mg/g GAE for the methanolic extract and 16.98 ± 0.45 mg/g GAE for the ethanolic leaf [54] 

There is a wide array of oxidants in the human body, comprising free radicals, reactive oxygen species (ROS), and reactive nitrogen species (NRS). Plant extracts or phytochemicals with a strong activity of those oxidants would be beneficial and of promising application potential. 

Phenolic compounds are the most recognizable plant-derived antioxidants. They exert their antioxidant activity via electron or hydrogen donation and metal reduction [55,56]. Such activity is attributed to their hydroxylation pattern [40]. Due the complexity of natural products [56] and their different reaction modes as antioxidants whether by SET, HAT, or transition-metal chelation [53,57], we estimated the in vitro antioxidant activity of *C. endivia* crude extract by applying three different antioxidant assays DPPH, FRAP, and phosphomolybdenum tests. The DPPH^●^ is a remarkable stable radical due to its delocalization in the aromatic rings. The antioxidant species neutralizes this radical by SET or HAT converting it into a reduced form (DPPH or DPPH-H) [53,58] resulting in its discoloration from intense purple to pale yellow. The DPPH assay is only applicable to lipophilic antioxidants [51,52,53]. On the one hand, the FRAP assay involves the reduction potential of transition metals, namely iron (Fe^+3^), and depends mainly on the SET mode [52,53], but occasionally could act through metal chelation [53]. The FRAP assay is suitable for hydrophilic and lipophilic samples [53], while the phosphomolybdenum assay for the estimation of TAC relies on the HAT or SET mechanism, where the antioxidant species reduces molybdenum (VI) to molybdenum (V) producing a greenish-blue complex. This greenish-blue color is formed as a consequence of ammonium molybdate reduction into an oxide [H_3_PO_4_(MoO_3_)_12_] known as the Keggin ion in acidic pH. Then, in the presence of an antioxidant compound, this Keggin ion is reduced into [H_4_PMo_8_VIMo_4_VO_40_]^3−^ [53]. A phosphomolybdenum assay has a wide range of screening since it is suitable for both fat-soluble and water-soluble antioxidants [53,59,60] and involves SET and HAT mechanisms. Moreover, this method is a reliable, optimized, and reproducible method to evaluate the total antioxidant capacity of many plant extracts and can be an alternative to HPLC methods [37].

Our results represented in Figure 2A–C demonstrated that *C. endivia* L. crude extract possessed notable DPPH radical scavenging activity with an IC_50_ value of 105.66 ± 4.98 µg/mL compared to the ascorbic acid reference standard with IC_50_ = 10.64 ± 0.82 µg/mL. Furthermore, the extract displayed its greatest scavenging activity at 1.28 mg/mL representing 92.34 ± 1.28% compared to 98.91 ± 0.74% for ascorbic acid at the same concentration. These findings are in agreement with Marzouk et al. [61]. Regarding the FRAP and phosphomolybdenum TAC assays, to the best of our knowledge, this is the first time those tests have been employed to determine the antioxidant activity of *C. endivia*. Our findings revealed that *C. endivia* crude extract displayed promising ferric ion reducing potential with IC_50_ = 125.71 ± 5.27 µg/mL in comparison to ascorbic acid (IC_50_ = 18.7 ± 1.26 µg/mL), indicating the electron-donating ability and linked to a lower generation of oxidant species; this is thought to be a key mechanism of phenolic compounds as antioxidant species [62,63]. Finally, the TAC assay indicated that *C. endivia* had a remarkable antioxidant potential with a value of 27.43 ± 2.09 mg GAE/g comparable to that of the reference standard, ascorbic acid (67.48 ± 3.14 mg GAE/g). Interestingly, the TPC value of *C. endivia* crude extract (26.41 ± 2.35 mg GAE/g) approached that of TAC (27.43 ± 2.09 mg GAE/g), suggesting that the antioxidant capacity of *C. endivia* is attributed mainly to its phenolic compounds constituting the major phytochemicals of the plant (evidenced by the detailed chromatographic investigation) rather than other chemical entities belonging to different chemical classes.

Among the isolated compounds, the phenolic compounds vanillic, ferulic, and *p*-coumaric acids were the major phytoconstituents of *C. endivia*, which were obtained in a considerable amount, sufficient for our in vivo study, as these compounds have been proved to have diverse activities and can be used as chemoprotective, anti-inflammatory, and antioxidant agents [64,65]. 

Encouraged by the aforesaid findings, we decided to prepare the phenolic fraction of *C. endivia* to be inspected for its gonado-protective effect along with the plant crude extract and the isolated acids, to confirm the medicinal value of *C. endivia* as well as of the isolated phenolic acid as economic, natural, safe, available, and applicable protective agents. The matter which opens a new horizon in overcoming the side effects associated with chemotherapy.

The chemotherapeutic MTX negatively affects the male reproductive system and can lead to sterility with long-term use. Several studies have revealed this gonadal toxicity in human and indicated oligospermia, an increased count of immature germinal cells, immobilization of sperms, and other alterations in semen parameters [66,67,68]. In this context, MTX is commonly used to induce testicular injury in animal models to investigate the potential gonado-protective agents [29]. Our results revealed that the MTX control group showed a significant reduction in testosterone, CAT, SOD, and Bcl-2, with a significant increase in MDA, NF-κB, TNF-*α*, IL-1*β*, IL-6, miR-29a, p53, and Bax compared to the normal control group. The reduced serum testosterone is due to MTX toxicity on the testes, and this was evidenced by the histopathological examination and the Johnsen score, which was 2 for this group.

Oxidative stress plays an essential role in MTX-induced gonadal injury by overwhelming the antioxidant capacity of the cells, which is reflected in the drop in the antioxidant enzymes CAT and SOD, and this makes the testicular cells more vulnerable to the released reactive oxygen species (ROS) and lipid peroxidation products such as MDA. This unfavorable highly oxidative environment can lead to male infertility and sterility with reduced semen parameters, and this also explains the reduced testosterone level in the MTX group [69]. Moreover, the inflammatory reactions play a crucial part in the pathogenesis of testicular injury caused by MTX and this was indicated by the increased level of inflammatory biomarkers [29]. The released ROS can trigger such an inflammatory response due to the accumulation of the immune cells at the site of injury leading to the upregulation of proinflammatory cytokine [70]. The upregulation of TNF-α stimulates multiple pathways that eventually activate NF-κB [71], which is a key regulator in the initiation of the inflammatory response, and which in turn upregulates the inflammatory markers including IL-1β and IL-6 [72]. Our current results are in agreement with other studies which have reported the oxidative and inflammatory mechanisms by which MTX induces gonadal toxicity [29,69,70,71,72,73].

Apoptosis is a programmed cell death, which is regulated by a group of proteins that can be proapoptotic, such as Bax, or antiapoptotic, such as Bcl-2, with a significant role of the tumor suppressor protein p53 [74]. MTX is a competitive inhibitor for dihydrofolate reductase resulting in the disruption of several metabolic pathways including nucleic acid synthesis and therefore can lead to the induction of apoptosis and cell death. In addition, the ROS-releasing property of MTX is also a main mechanism by which apoptosis can be induced [28]. A study reported that p53 was an essential transcription factor for MTX-induced apoptosis [75]. Our present findings are consistent with others that reported the ability of MTX to upregulate p53 and Bax and downregulate Bcl-2 to induce apoptosis [28,29,70].

Among the miR-29 family, miR-29a has a proapoptotic activity. Some researchers reported that the miR-29a apoptotic effect was mediated through the PTEN/PI3K/Akt/GSK3β signaling pathway [76,77]. Another study figured out its apoptosis-inducing activity was p53-dependent [78]. Our results are in agreement with those of others who concluded that doxorubicin [79] and estradiol benzoate [80] induced gonadal apoptosis through the upregulation of miR-29a, and with another study that also used MTX to induce testicular injury and found that miR-29a had an apoptotic activity [29].

Since oxidative stress, inflammation, and apoptosis are the main mechanisms by which MTX induces toxicity and causes the loss of spermatocytes, as well as the atrophy of seminiferous tubules [70], agents that have antioxidant, anti-inflammatory, and antiapoptotic effects would be beneficial to ameliorate such germ cell toxicity. Many plants are the main sources for bioactive compounds that can be used to relieve diseases [81]. For instance, phytomedicine and plant-derived antioxidants are considered a prosperous strategy for alleviating chemotherapy-induced organ toxicities [28,82,83,84,85]. As a result, we aimed to evaluate the possible testiculo-protective effect of *C. endivia* L. crude extract, phenolic fraction, and isolated coumaric, vanillic, and ferulic acids against MTX-induced testicular injury in mice. A pretreatment with *C. endivia* L. and the isolated compounds restored the disorganized architecture of the testes to nearly normal, as indicated by the histopathology and Johnsen score and thereby significantly elevated the serum level of testosterone compared to MTX alone. Our results are in agreement with those of others who suggested the useful use of *Cichorium intybus*, another species of the same family as *C. endivia* L., in testicular injury [86,87] and with others who examined the protective effect of coumaric [88], vanillic [89,90], and ferulic acids [89,90,91] against testicular damage.

The antioxidant activity of *C. endivia* L. was revealed by the increased level of CAT and SOD with the decreased level of MDA. This could be attributed to the phenolic compounds that represent the major antioxidants because of their hydrogen-donating character and their ability to prohibit membrane lipid peroxidation, thereby causing membrane stabilization and limiting the passage of the free radicals [87]. Our findings are in agreement with another study that investigated the antioxidative action of *C. endivia* L. extract against hepatotoxicity induced by tert-butyl hydroperoxide in vitro and in vivo [12]. Moreover, other studies documented the improved oxidative status of male reproductive organs by *C. intybus* [86,87]. Coumaric [92], vanillic [89,90,93,94], and ferulic acids [89,91,95,96] are phenolic compounds which are widely dispersed in numerous plants and have been extensively investigated for their antioxidant property. In the same manner, *C. endivia* L. crude extract and the phenolic fraction demonstrated an anti-inflammatory action by decreasing NF-κB, IL-1*β*, IL-6, and TNF-α. Our results are in agreement with clinical trials that used *C. intybus* extract and proved its anti-inflammatory effect against different inflammatory conditions [97,98] and in an experimental model for diabetes [99]. The presence of phenolic compounds may also be responsible for that anti-inflammatory effect, as indicated by previous studies [100,101]. Coumaric acid demonstrated its anti-inflammatory activity by lowering the proinflammatory cytokines and decreasing the expression of NF-κB, and this is compatible with results from Sabitha et al. [102] and Abdel-Moneim et al. [103]. Vanillic acid also demonstrated an anti-inflammatory effect, which is in agreement with other studies indicating the same results [94,104,105]. Hu et al. [104], Hassanein et al. [91], and Kassab et al. [95] reported the anti-inflammatory action of ferulic acid and this supports our results.

The gonado-protective effect of *C. endivia* L. was also mediated through antiapoptotic mechanism, which was reflected by the suppressed testicular proapoptotic Bax, as well as the tumor suppressor p53 with an increase in the antiapoptotic Bcl-2 level. This is in agreement with Elmasry et al. [106] and Erkec et al. [107], who revealed the antiapoptotic action of *C. intybus* extract. The antiapoptotic effect of the phenolic fraction is consistent with other earlier studies that reported the antiapoptotic effect of phenolic compounds [108,109]. Regarding the effect of the isolated compounds, our findings are supported by others that demonstrated the antiapoptotic effect of coumaric [102,103], vanillic [90,110], and ferulic [90,95] acids in different experimental animal models.

It has been suggested that the lipid peroxidation, oxidative stress, and reduced antioxidant capacity are associated with the upregulation of miR-29a [111]. miR-29a is also strongly linked to the inflammatory response through several mechanisms including the increased phosphorylation of P65, serine/threonine kinase 1 (Akt1), and NF-κB signaling pathways [112]. Additionally, the upregulated miR-29a can increase the production of IL-1β and IL-6 through triggering NF-κB [112]. Therefore, agents that possess antioxidant and anti-inflammatory effects can downregulate miR-29a. *C. endivia* L. crude extract, the phenolic fraction, and the isolated compounds successfully downregulated testicular miR-29a and thereby enhanced MTX-induced gonadal injury. Our results are in line with other studies indicating the inhibitory effect of various natural products against different miRNAs [29,113,114].

## 5. Conclusions

Herein, a phytochemical investigation of *Cichorium endivia* L. led to the isolation of 13 compounds: stigmasterol, ursolic acid, *β*-amyrin, azelaic acid, vanillic acid, (6S, 7E)-6-hydroxy-4,7-megastigmadien-3,9-dione (S(+)-dehydrovomifoliol), 4-hydroxy phenyl acetic acid, vomifoliol, ferulic acid, protocatechuic acid, kaempferol, *p*-coumaric acid and luteolin. The total extract was enriched with phenolic contents as shown with the TPC assay and it exhibited a marked antioxidant activity via DPPH, FRAP, and TAC assays. *C. endivia* L. crude extract, its phenolic fraction, and the isolated coumaric, vanillic, and ferulic acids exhibited a protective effect against MTX-induced germ cell toxicity through anti-inflammatory, antioxidant, and antiapoptotic effects. These beneficial outcomes suggest *C. endivia* L. can be used in further studies to investigate more molecular mechanisms underlying its pharmacological activity.

## Figures and Tables

**Table 1:** Primer sequences and annealing temperatures for the measured genes.

	Forward Primer	Reverse Primer	Annealing Temperature
NF-κb	5′-CAATGGCTACACAGGACCA-3′	5′-CACTGTCACCTGGAACCAGA-3′	52 °C
TNF-α	5′-TCTACTGAACTTCGGGGTGATCG-3′	5′-TGATCTGAGTGTGAGGGTCTGGG-3′	56 °C
p53	5′-ACCGCCGACCTATCCTTACC-3′	5′-TCTTCTGTACGGCGGTCTCTC-3′	56 °C
β-actin	5′-ACGGCCAGGTCATCACTATTG-3′	5′-CAAGAAGGAAGGCTGGAAAAGA-3′	52 °C
miR-29a	5′-GCGCACTGATTTCTTTTGGTGTTCAG-3′	5′-GCGAGCACAGAATTAATACGAC-3′	51 °C
RNU6B	5′-CTCGCTTCGGCAGCACATA-3′	5′-CGCTTCACGAATTTGCGTG-3′	53 °C

NF-κB: nuclear factor kappa B; TNF-α: tumor necrosis factor alpha; RNU6B: U6B small nuclear RNA.

## Data Availability

Data are available within the article and Supplementary Materials.

## Supplementary Materials

The following supporting information can be downloaded at: https://www.mdpi.com/article/10.3390/biomedicines10081986/s1, Figures S1 and S2: ^1^H NMR,^13^C NMR spectra of compound **1**, Figures S3 and S4: ^1^H NMR,^13^C NMR spectra of compound **4**, Figures S5–S7: ^1^H NMR,^13^C NMR, and ^13^C NMR APT spectra of compound **5**, Figures S8–S12: ^1^H NMR,^13^C NMR, ^13^C NMR APT, HSBC, and HMBC spectra of compound **6**, Figures S13–S15: ^1^H NMR,^13^C NMR, and ^13^C NMR APT spectra of compound **7**, Figures S16–S20: ^1^H NMR,^13^C NMR, ^13^C NMR APT, HSBC, and HMBC spectra of compound **8**, Figures S21–S23: ^1^H NMR,^13^C NMR, and ^13^C NMR APT spectra of compound **9**, Figures S24–S28: ^1^H NMR,^13^C NMR, ^13^C NMR APT, HSBC, and HMBC spectra of compound **10**, Figures S29–S31: ^1^H NMR,^13^C NMR, and ^13^C NMR APT spectra of compound **12**.
