# Supplementary material for: Potential Gonado-Protective Effect of Cichorium endivia and Its Major Phenolic Acids against Methotrexate-Induced Testicular Injury in Mice
Source: Biomedicines. 2022 Aug 16;10(8):1986. doi: 10.3390/biomedicines10081986 (PMC9406180; doi:10.3390/biomedicines10081986)
Supplement: Supplementary file 1 [file biomedicines-10-01986-s001.zip › biomedicines-1805047-supplementary.pdf]

Potential Gonado-Protective Effect of *Cichorium endivia* and Its Major Phenolic Acids against Methotrexate-Induced Testicular Injury in Mice

Enas E. Eltamany [1], Esraa M. Mosalam [2], Eman T. Mehanna [3], Basma M. Awad [4], Sarah M. Mosaad [5], Maged S. Abdel-Kader [6,7,*], Amany K. Ibrahim [1], Jihan M. Badr [1] and Marwa S. Goda [1]

[1] Department of Pharmacognosy, Faculty of Pharmacy, Suez Canal University, Ismailia 41522, Egypt
[2] Department of Biochemistry, Faculty of Pharmacy, Menoufia University, Shebin El-Koum 32511, Egypt
[3] Department of Biochemistry, Faculty of Pharmacy, Suez Canal University, Ismailia 41522, Egypt
[4] Department of Pharmacognosy, Faculty of Pharmacy, Sinai University, El-Arish 45518, Egypt
[5] Division of Pharmacology and Therapeutics, Department of Continuous Medical Education, General Authority of Healthcare, Ismailia 41522, Egypt
[6] Department of Pharmacognosy, College of Pharmacy, Prince Sattam Bin Abdulaziz University, Al-Kharj 11942, Saudi Arabia
[7] Department of Pharmacognosy, Faculty of Pharmacy, Alexandria University, Alexandria 21215, Egypt
* Correspondence: m.youssef@psau.edu.sa; Tel.: +966-545-539-145

Figure No.	Title	Page
Figure S1:	^{1}H NMR spectrum of compound **(1)** Stigmasterol.	4
Figure S2:	^{13}C NMR spectrum of compound **(1)** Stigmasterol.	5
Figure S3:	^{1}H NMR spectrum of compound **(4)** Azelaic acid.	6
Figure S4:	^{13}C NMR spectrum of compound **(4)** Azelaic acid.	7
Figure S5:	^{1}H NMR spectrum of compound **(5)** Vanillic acid.	8
Figure S6:	^{13}C NMR spectrum of compound **(5)** Vanillic acid.	9
Figure S7:	^{13}C NMR APT spectrum of compound **(5)** Vanillic acid.	10
Figure S8:	^{1}H NMR spectrum of compound **(6)** (6S, 7E)-6-hydroxy-4,7-megastigmadien-3,9-dione (S(+)-dehydrovomifoliol).	11
Figure S9:	^{13}C NMR spectrum of compound **(6)** (6S, 7E)-6-hydroxy-4,7-megastigmadien-3,9-dione (S(+)-dehydrovomifoliol).	12
Figure S10:	^{13}C NMR APT spectrum of compound **(6)** (6S, 7E)-6-hydroxy-4,7-megastigmadien-3,9-dione (S(+)-dehydrovomifoliol).	13
Figure S11:	HSBC NMR spectrum of compound (**6)** (6S, 7E)-6-hydroxy-4,7-megastigmadien-3,9-dione (S(+)-dehydrovomifoliol).	14
Figure S12:	HMBC NMR spectrum of compound (**6)** (6S, 7E)-6-hydroxy-4,7-megastigmadien-3,9-dione (S(+)-dehydrovomifoliol).	15
Figure S13:	^{1}H NMR spectrum of compound **(7)** 4-Hydroxy phenyl acetic acid.	16
Figure S14:	^{13}C NMR spectrum of compound **(7)** 4-Hydroxy phenyl acetic acid.	17
Figure S15:	^{13}C APT NMR spectrum of compound **(7)** 4-Hydroxy phenyl acetic acid.	18
Figure S16:	^{1}H NMR spectrum of compound **(8)** Vomifoliol**.**	19
Figure S17:	^{13}C NMR spectrum of compound **(8)** Vomifoliol.	20

Figure S18:	^{13}C APT NMR spectrum of compound **(8)** Vomifoliol.	21
Figure S19:	HSBC NMR spectrum of compound **(8)** Vomifoliol.	22
Figure S20:	HMBC NMR spectrum of compound **(8)** Vomifoliol.	23
Figure S21:	^{1}H NMR spectrum of compound **(9)** Ferulic acid.	24
Figure S22:	^{13}C NMR spectrum of compound **(9)** Ferulic acid.	25
Figure S23:	^{13}C NMR APT spectrum of compound **(9)** Ferulic acid.	26
Figure S24:	^{1}H NMR spectrum of compound **(10)** Protocatechuic acid.	27
Figure S25:	^{13}C NMR spectrum of compound **(10)** Protocatechuic acid.	28
Figure S26:	^{13}C NMR APT spectrum of compound **(10)** Protocatechuic acid.	29
Figure S27:	HSBC NMR spectrum of compound **(10)** Protocatechuic acid.	30
Figure S28:	HMBC NMR spectrum of compound **(10)** Protocatechuic acid.	31
Figure S29:	^{1}H NMR spectrum of compound **(12)** *p*- Coumaric acid.	32
Figure S30:	^{13}C NMR spectrum of compound **(12)** *p*- Coumaric acid.	33
Figure S31:	^{13}C APT NMR spectrum of compound **(12)** *p*- Coumaric acid.	34

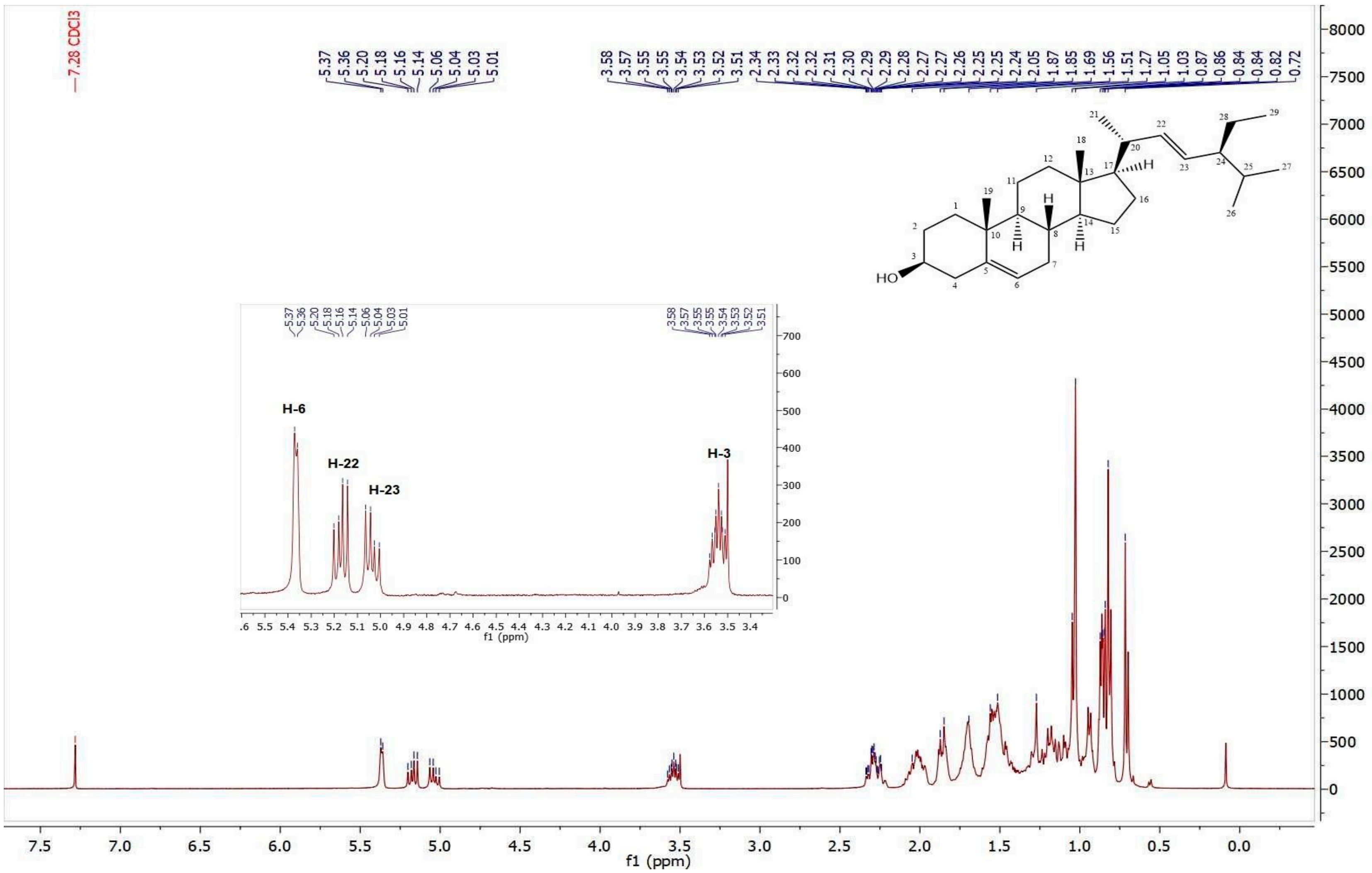

Figure S1: ^{1}H NMR spectrum of compound **(1)** Stigmasterol.

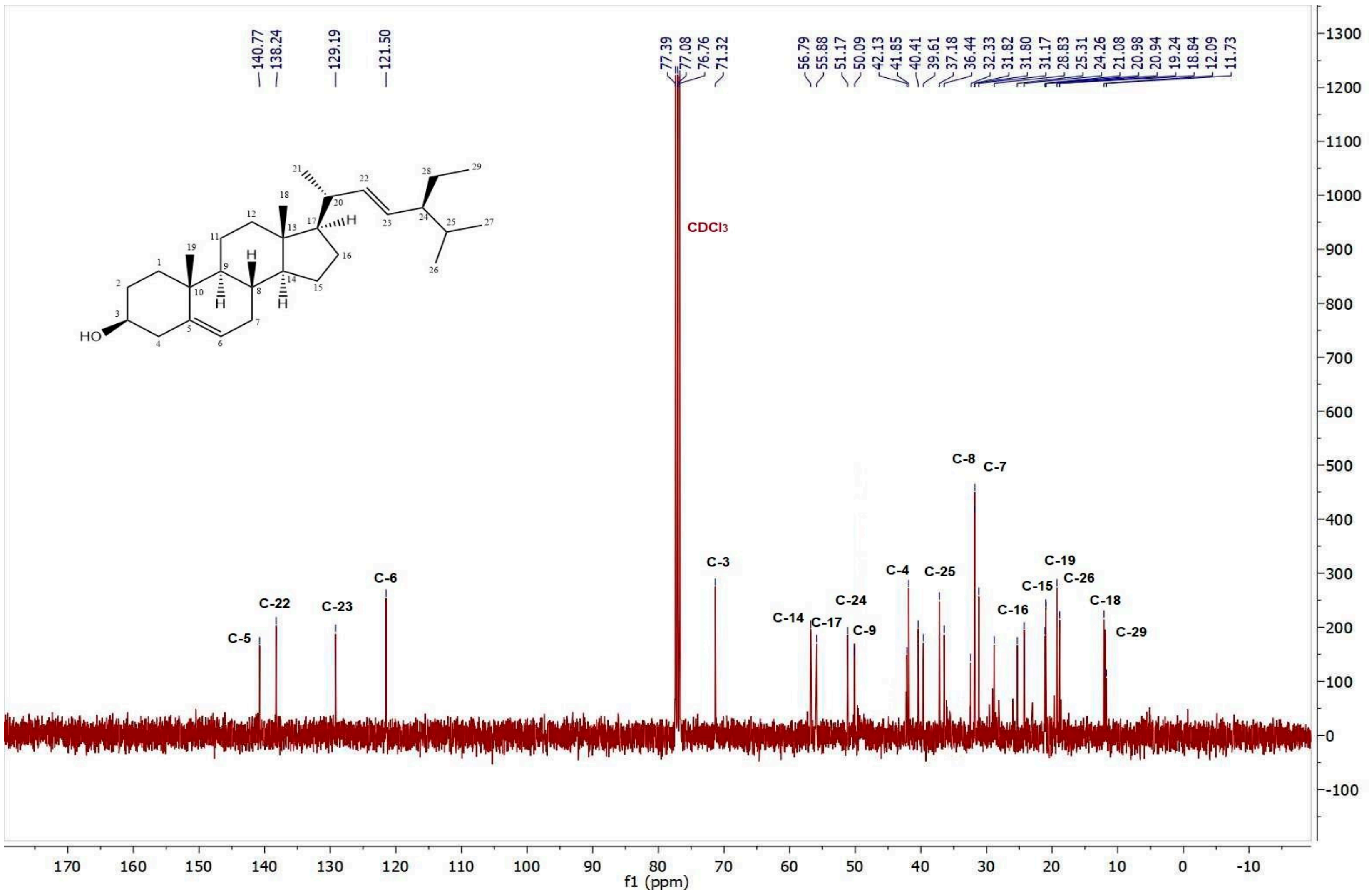

Figure S2: ^{13}C NMR spectrum of compound **(1)** Stigmasterol.

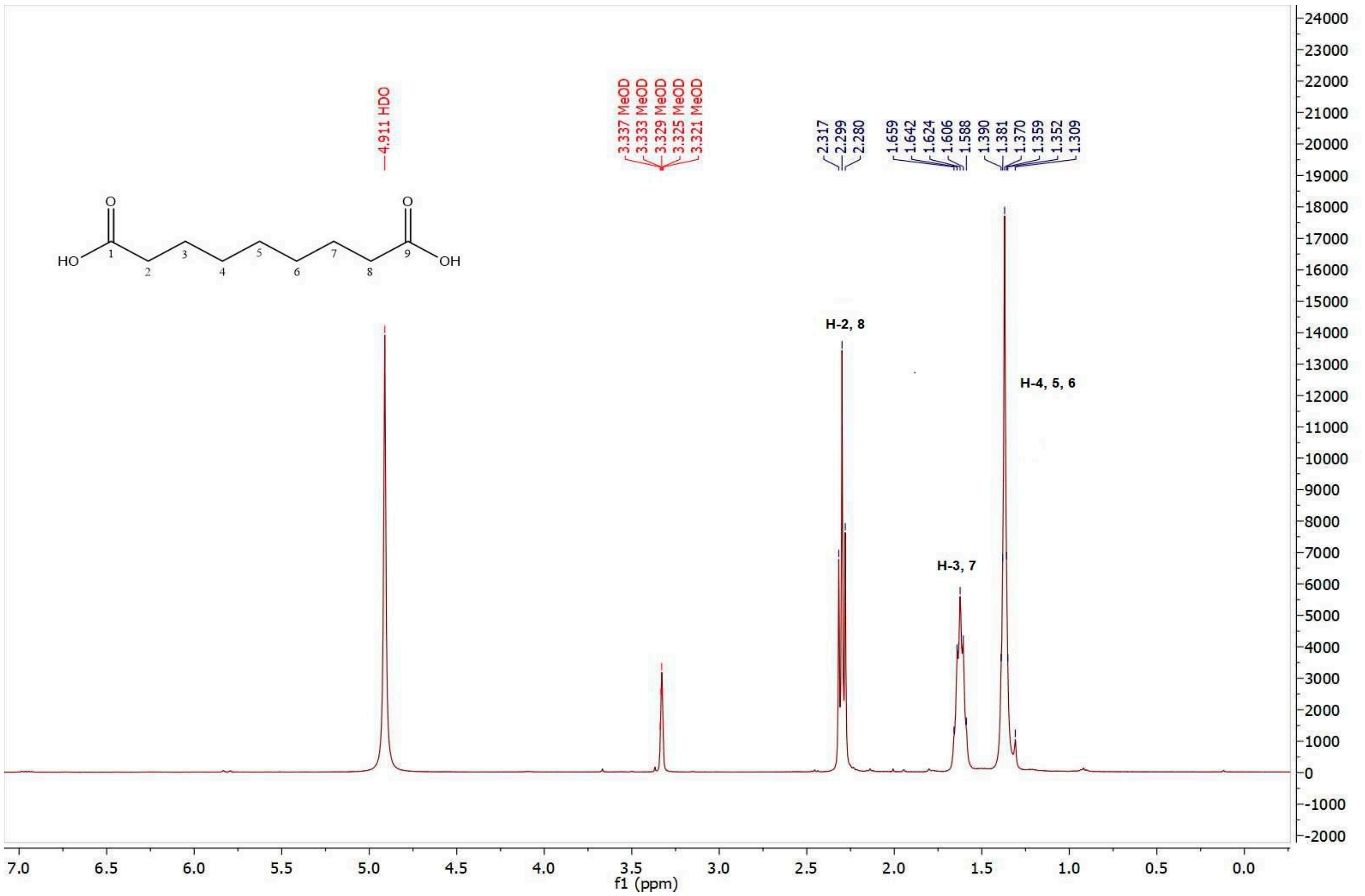

Figure S3: ^{1}H NMR spectrum of compound **(4)** Azelaic acid.

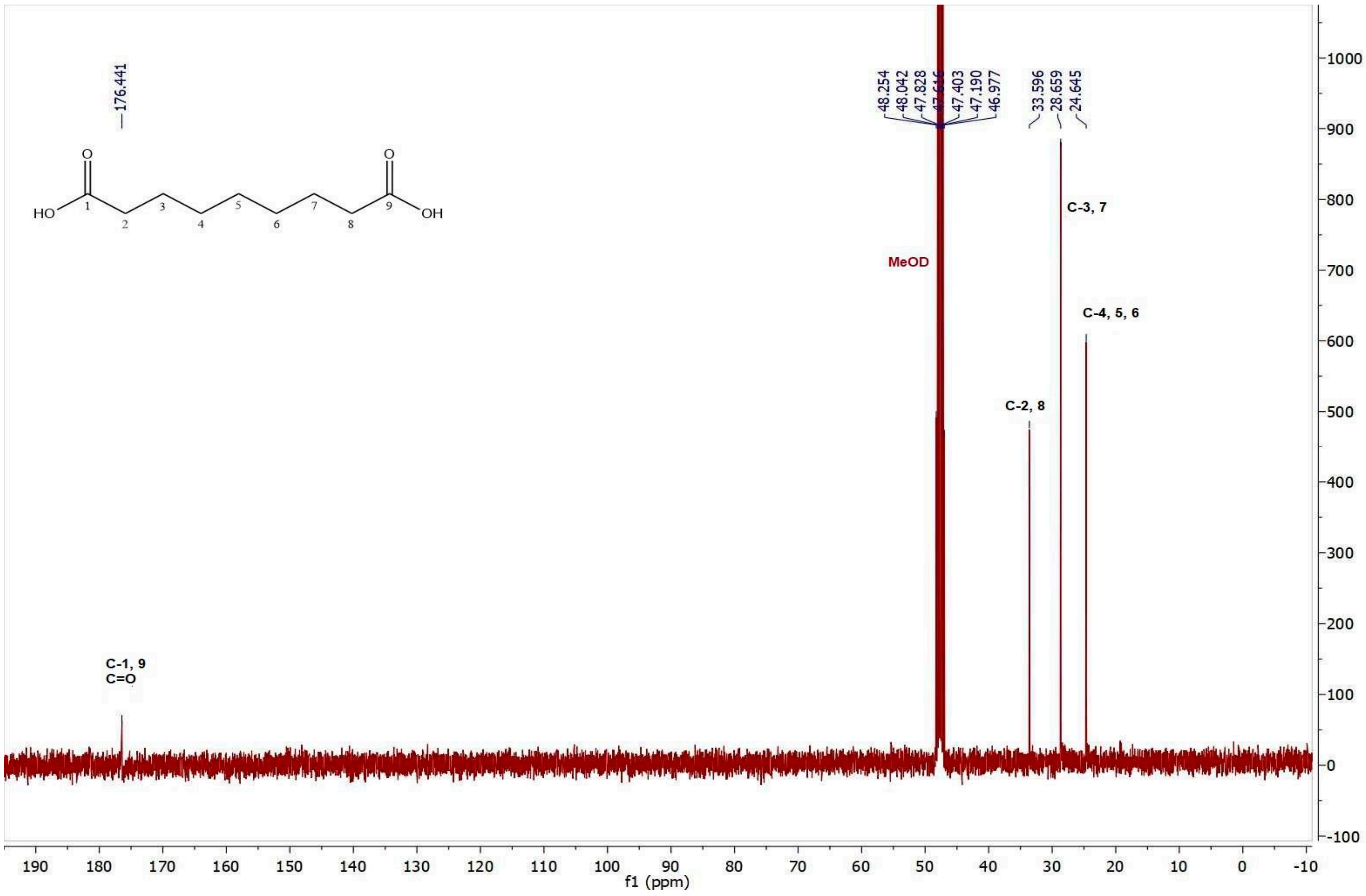

Figure S4: ^{13}C NMR spectrum of compound **(4)** Azelaic acid.

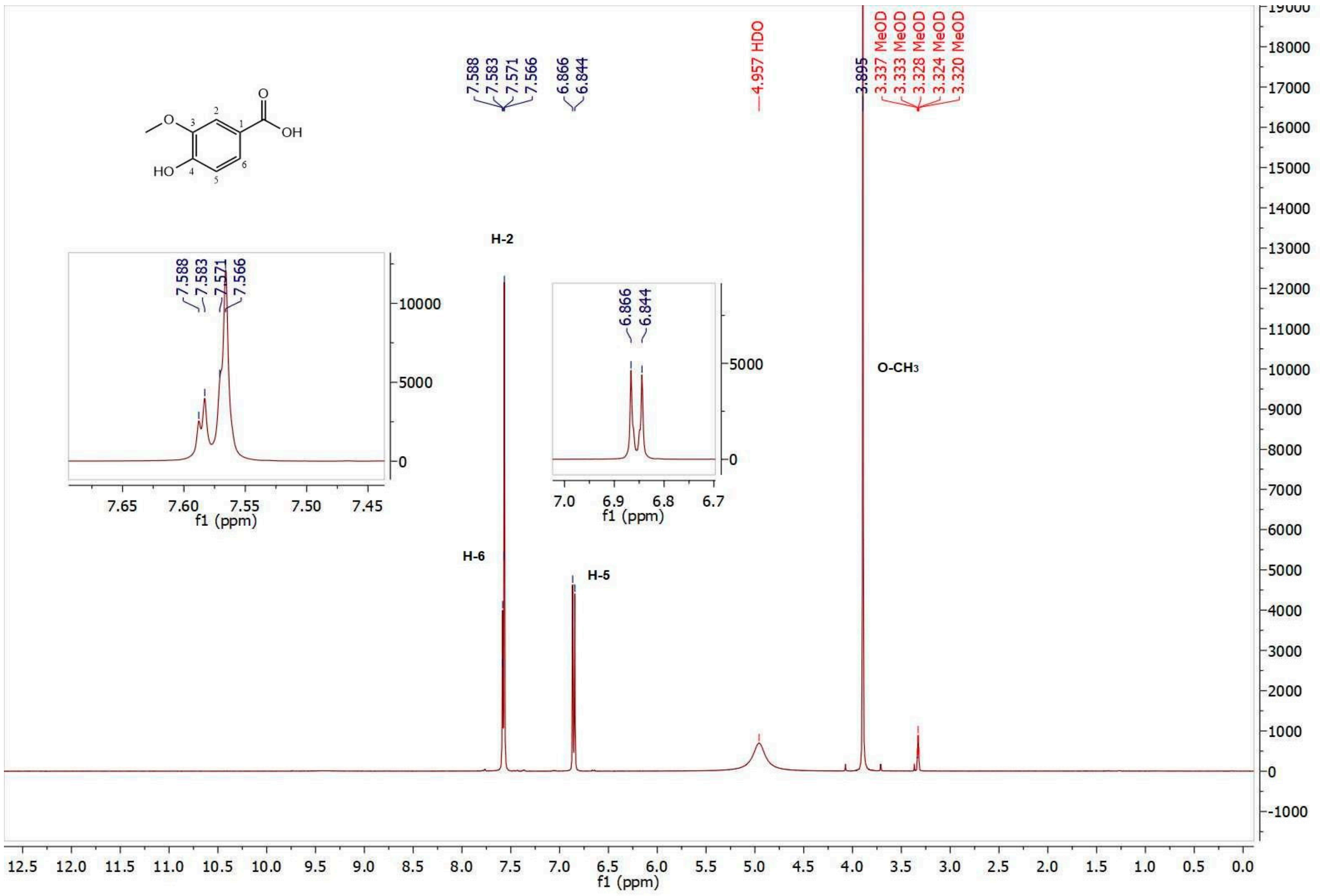

Figure S5: ^{1}H NMR spectrum of compound **(5)** Vanillic acid.

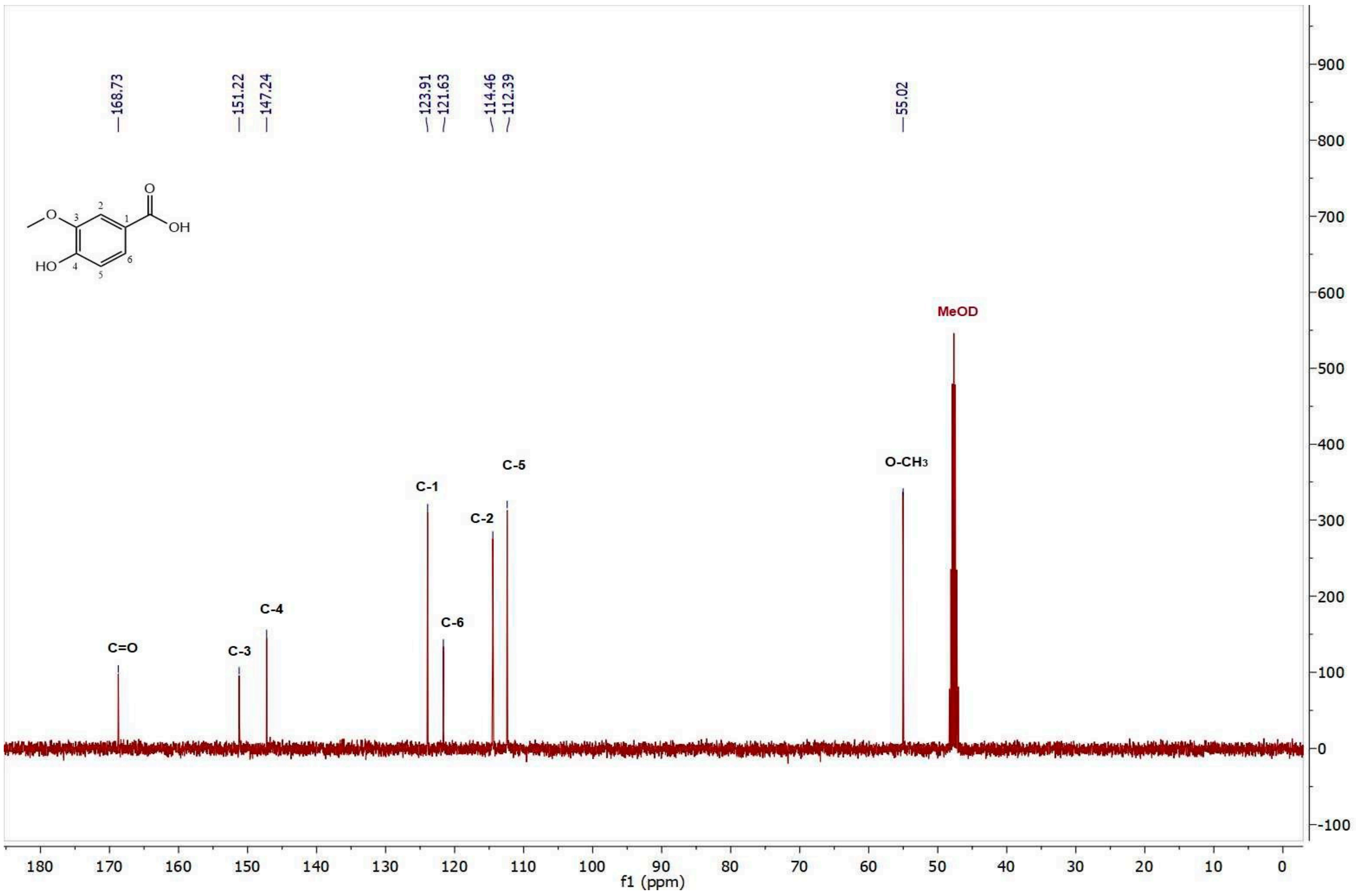

Figure S6: ^{13}C NMR spectrum of compound **(5)** Vanillic acid.

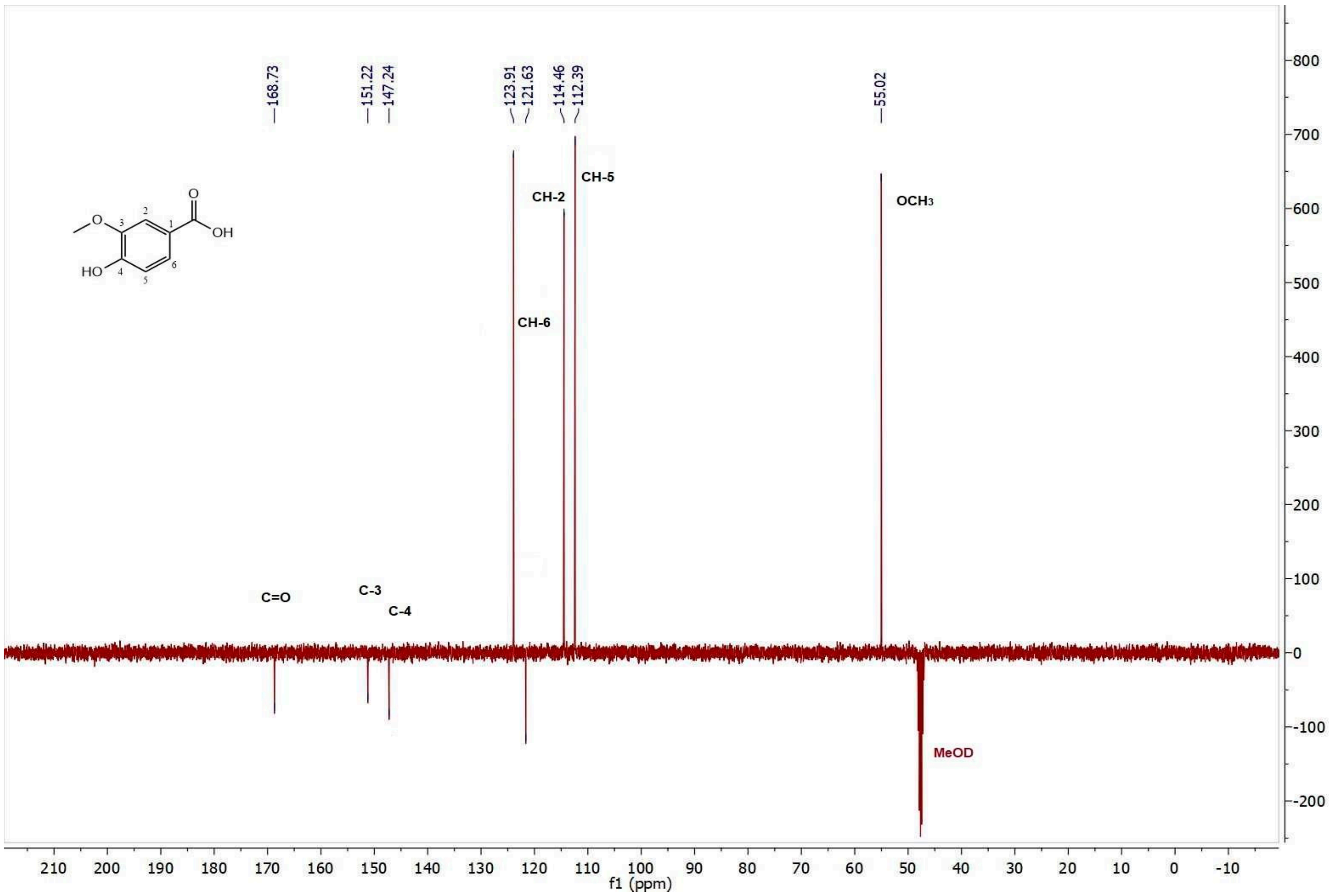

Figure S7: ^{13}C NMR APT spectrum of compound **(5)** Vanillic acid.

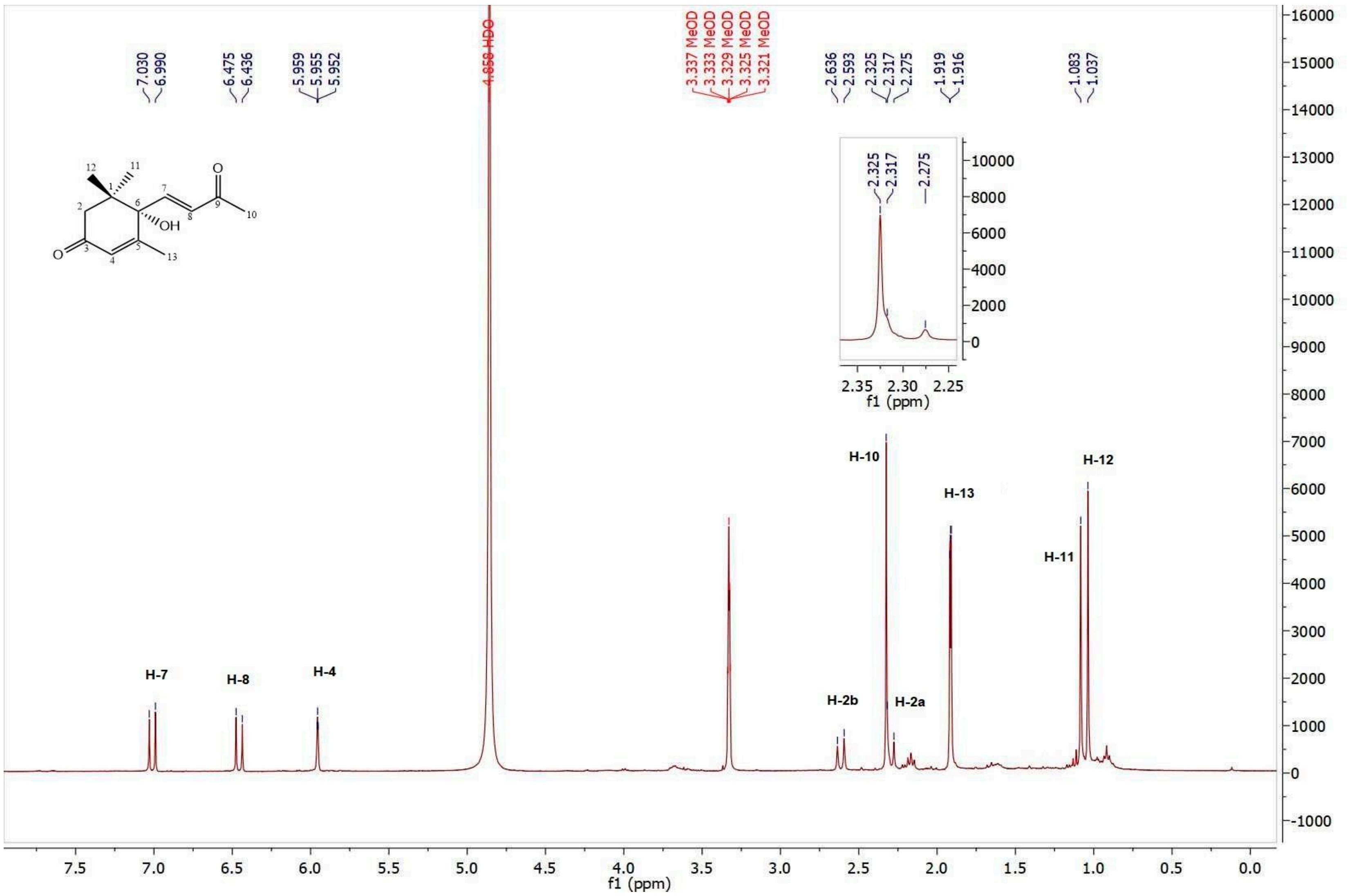

Figure S8: ¹H NMR spectrum of compound **(6)** (6S, 7E)-6-hydroxy-4,7-megastigmadien-3,9-dione (S(+)-dehydrovomifoliol).

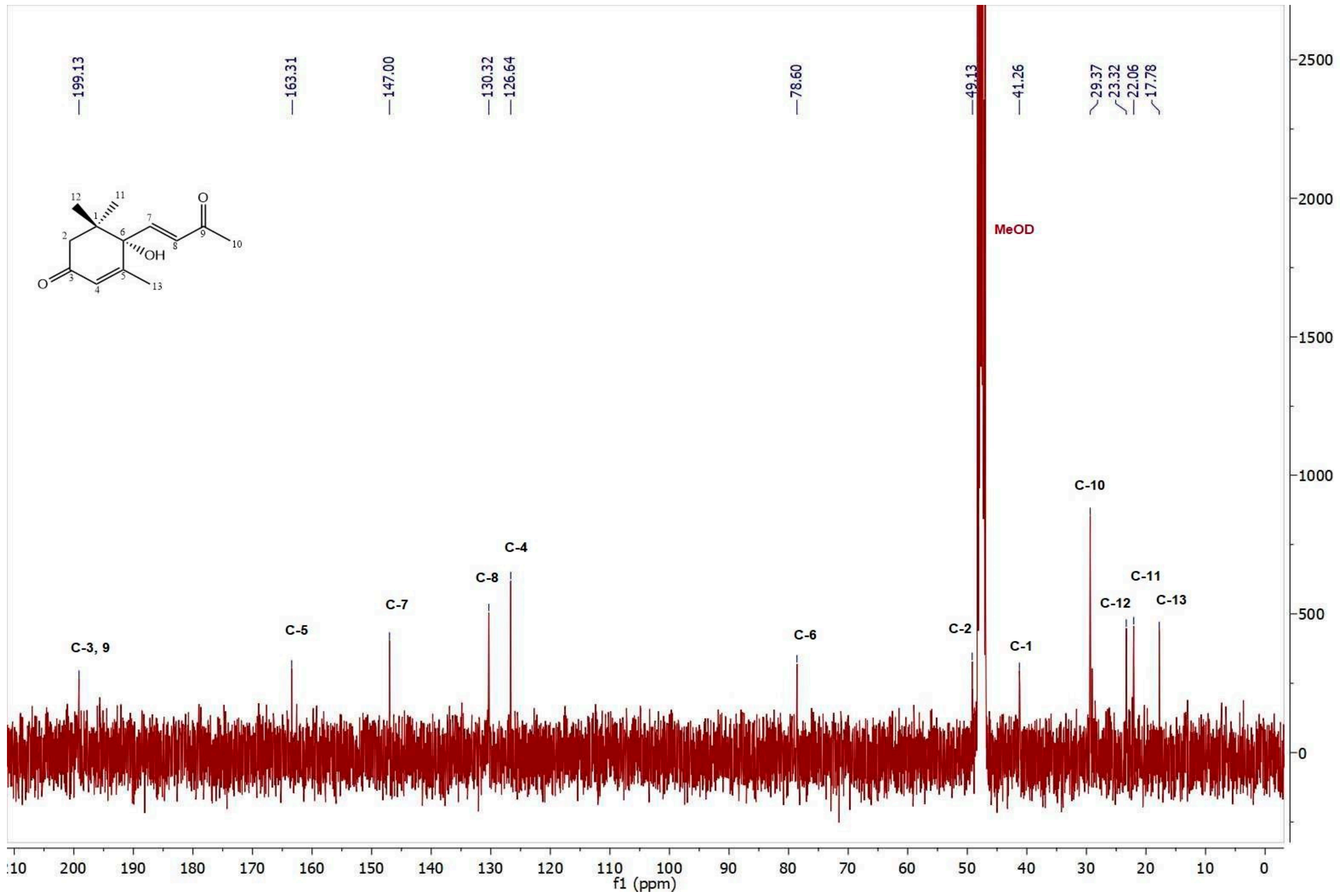

Figure S9: ^{13}C NMR spectrum of compound **(6)** (6S, 7E)-6-hydroxy-4,7-megastigmadien-3,9-dione (S(+)-dehydrovomifoliol).

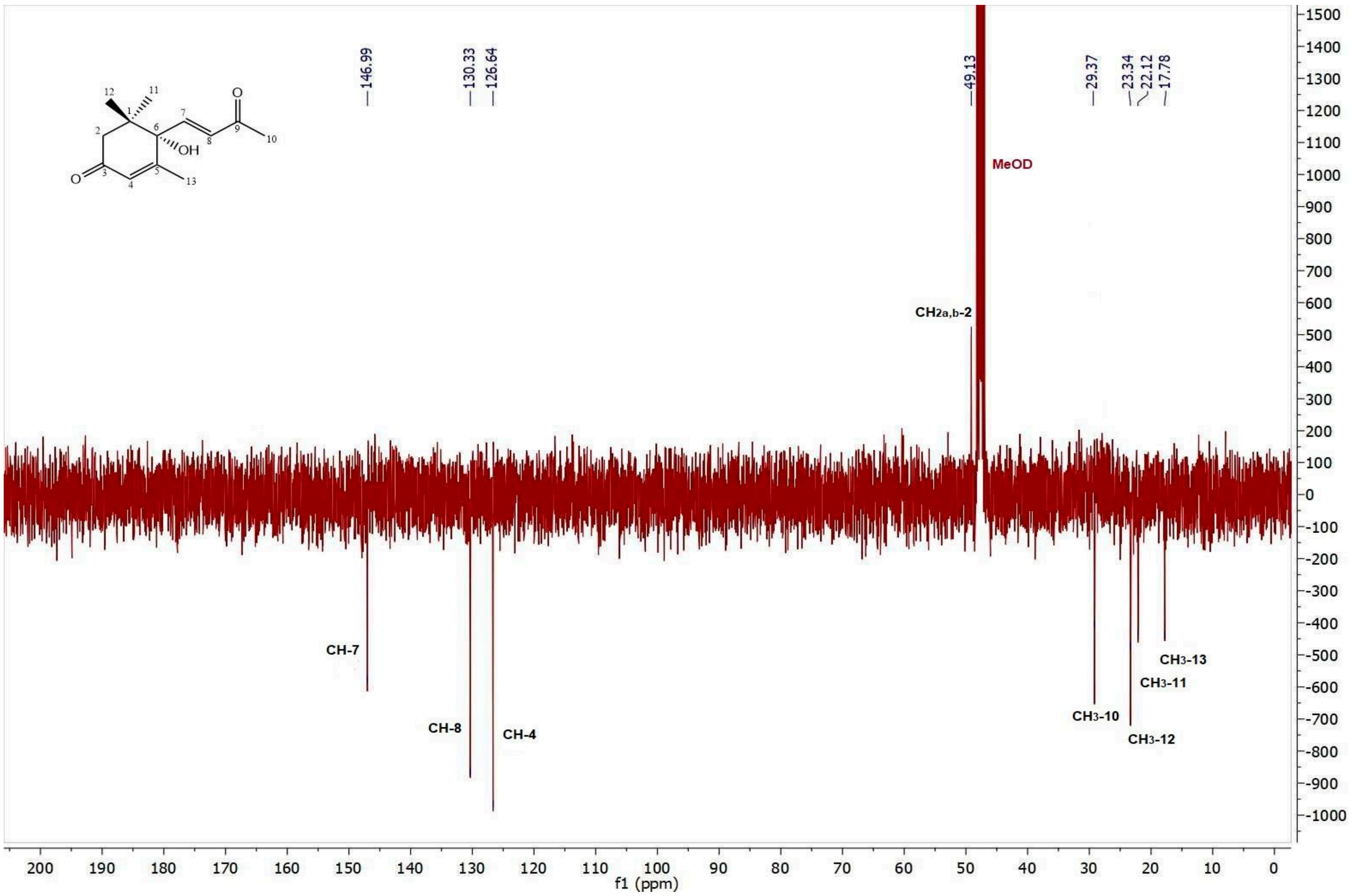

Figure S10: ^{13}C NMR APT spectrum of compound **(6)** (6S, 7E)-6-hydroxy-4,7-megastigmadien-3,9-dione (S(+)-dehydrovomifoliol).

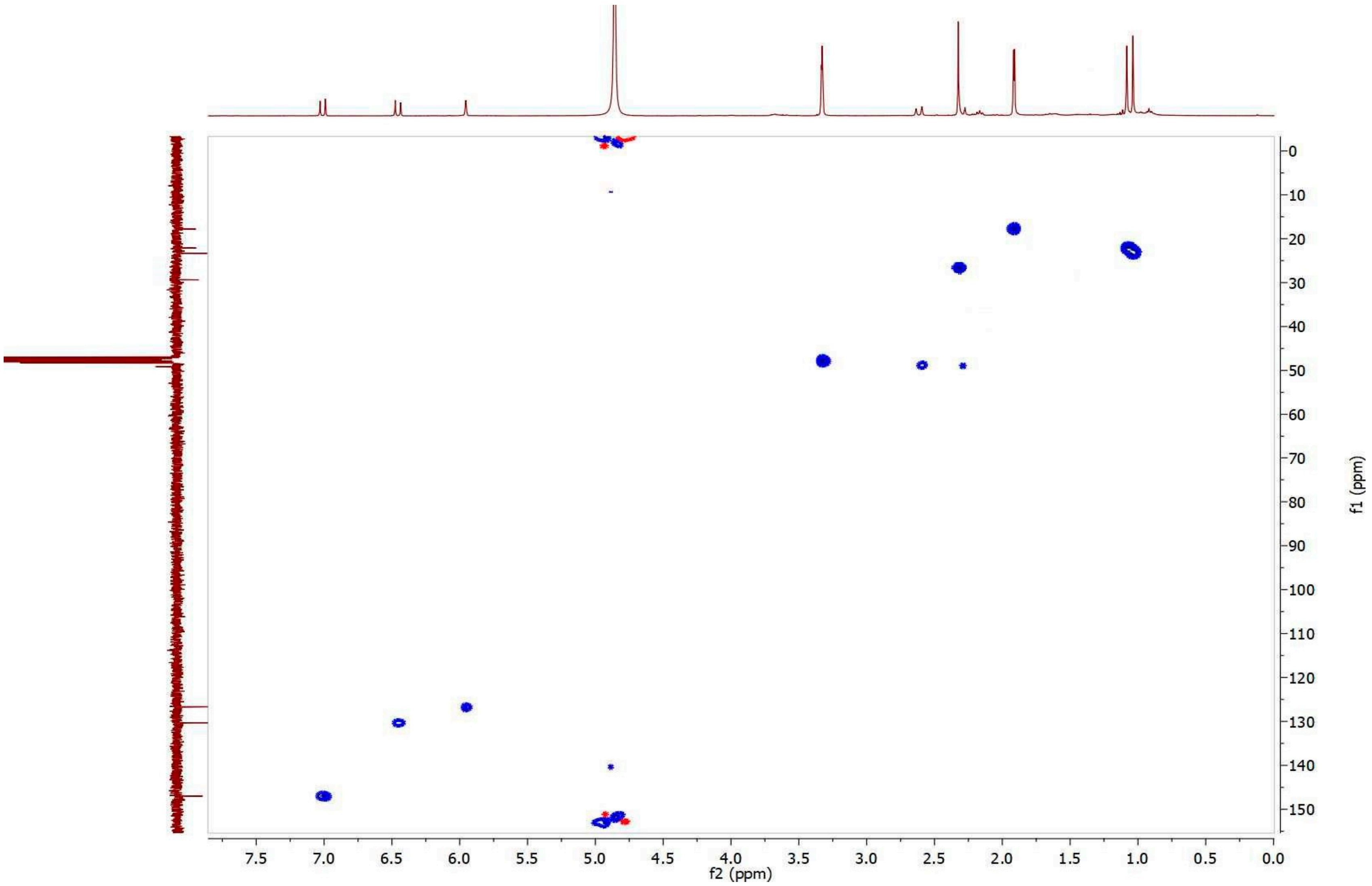

Figure S11: HSBC NMR spectrum of compound (**6)** (6S, 7E)-6-hydroxy-4,7-megastigmadien-3,9-dione (S(+)-dehydrovomifoliol).

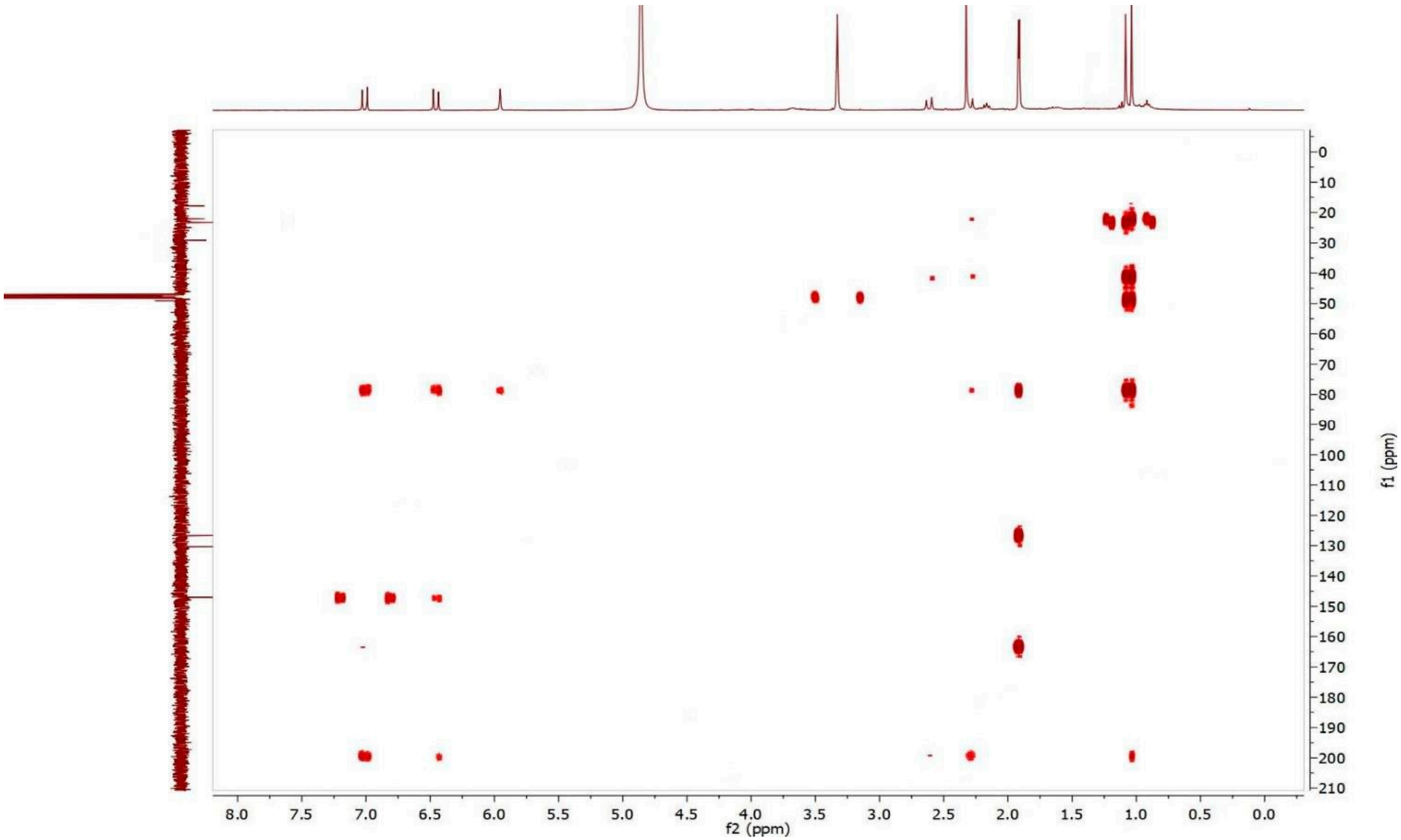

Figure S12: HMBC NMR spectrum of compound (**6**) (6S, 7E)-6-hydroxy-4,7-megastigmadien-3,9-dione (S(+)-dehydrovomifoliol).

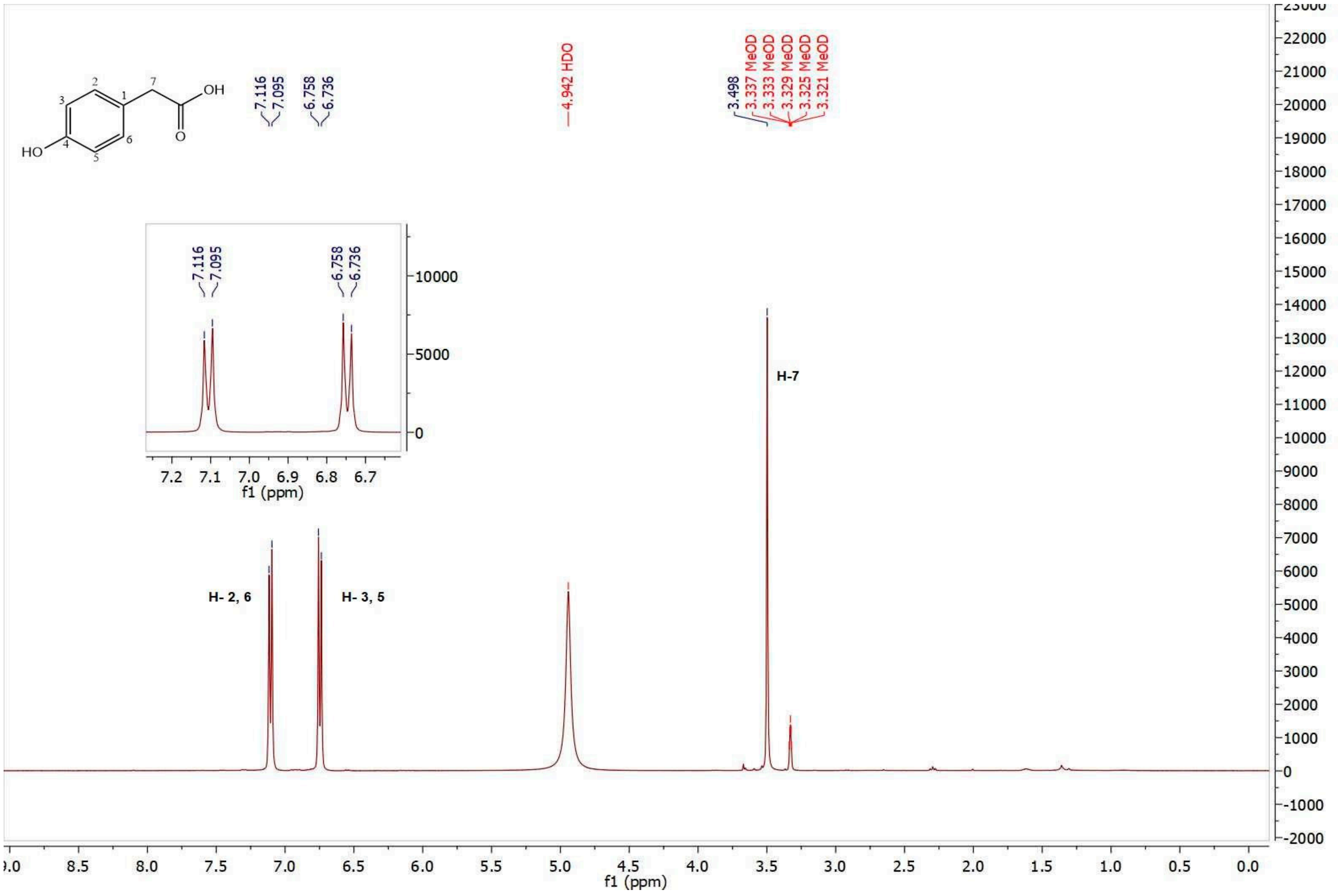

Figure S13: ^{1}H NMR spectrum of compound **(7)** 4-Hydroxy phenyl acetic acid.

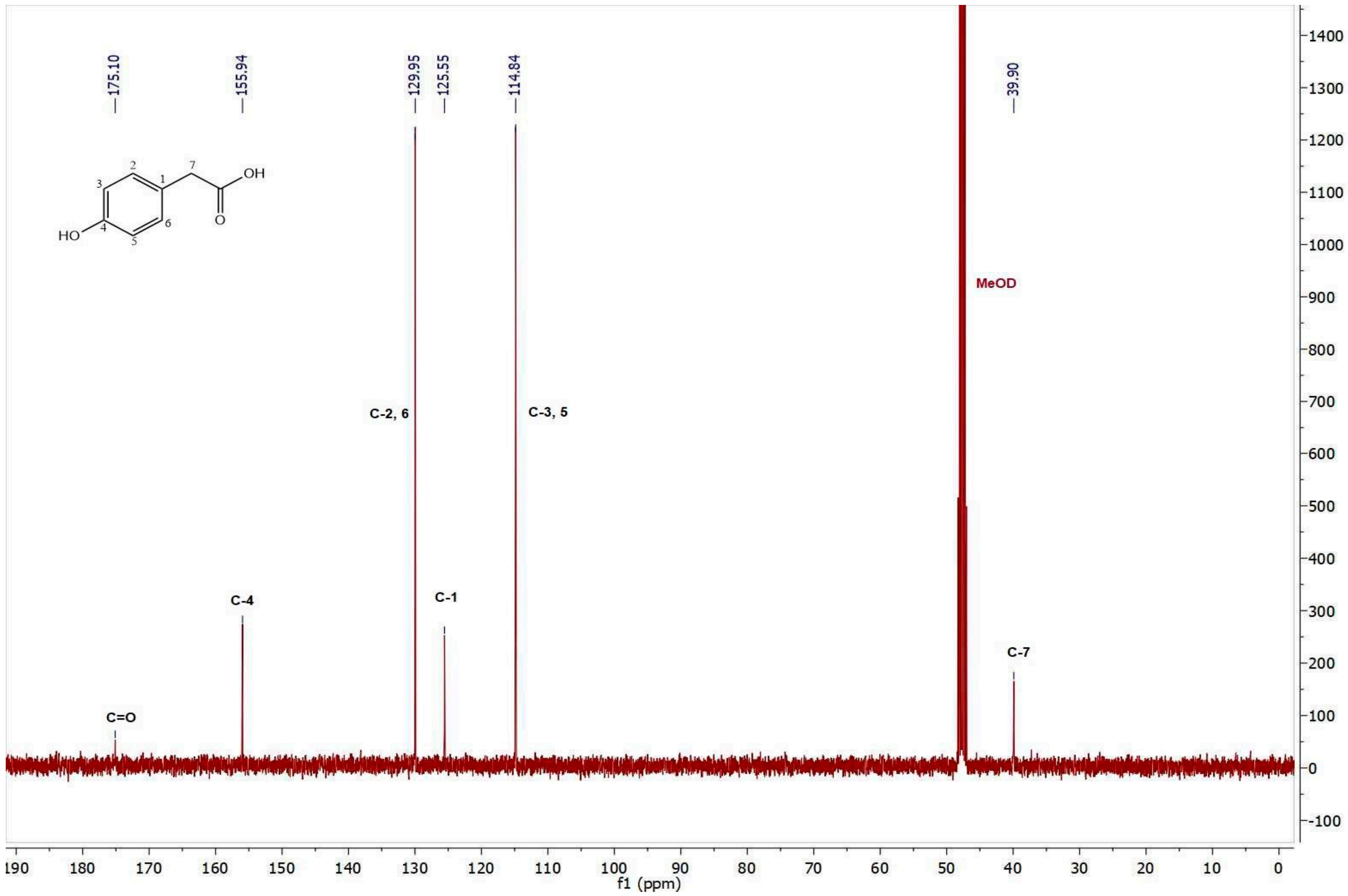

Figure S14: ^{13}C NMR spectrum of compound **(7)** 4-Hydroxy phenyl acetic acid.

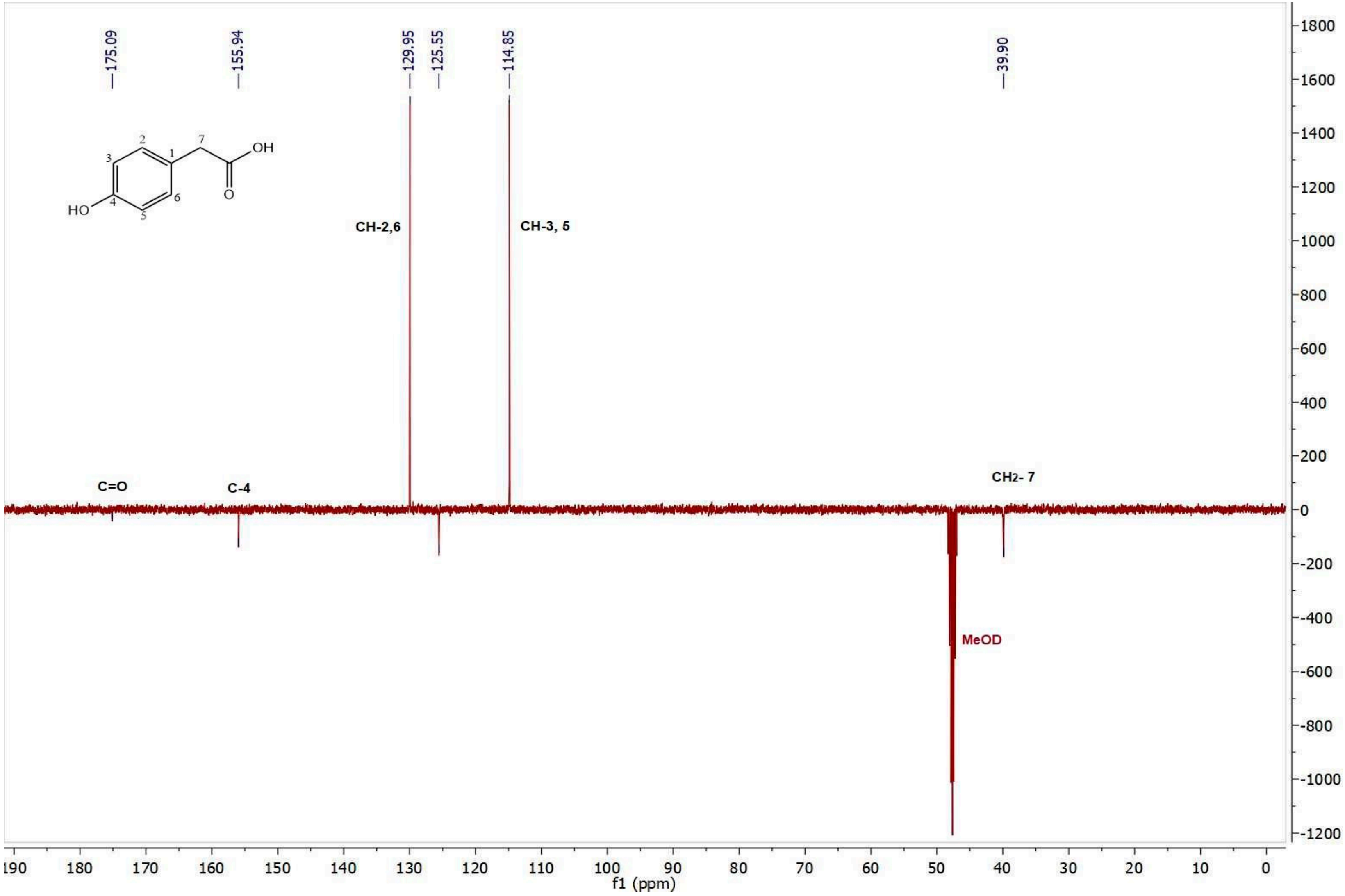

Figure S15: ^{13}C APT NMR spectrum of compound **(7)** 4-Hydroxy phenyl acetic acid.

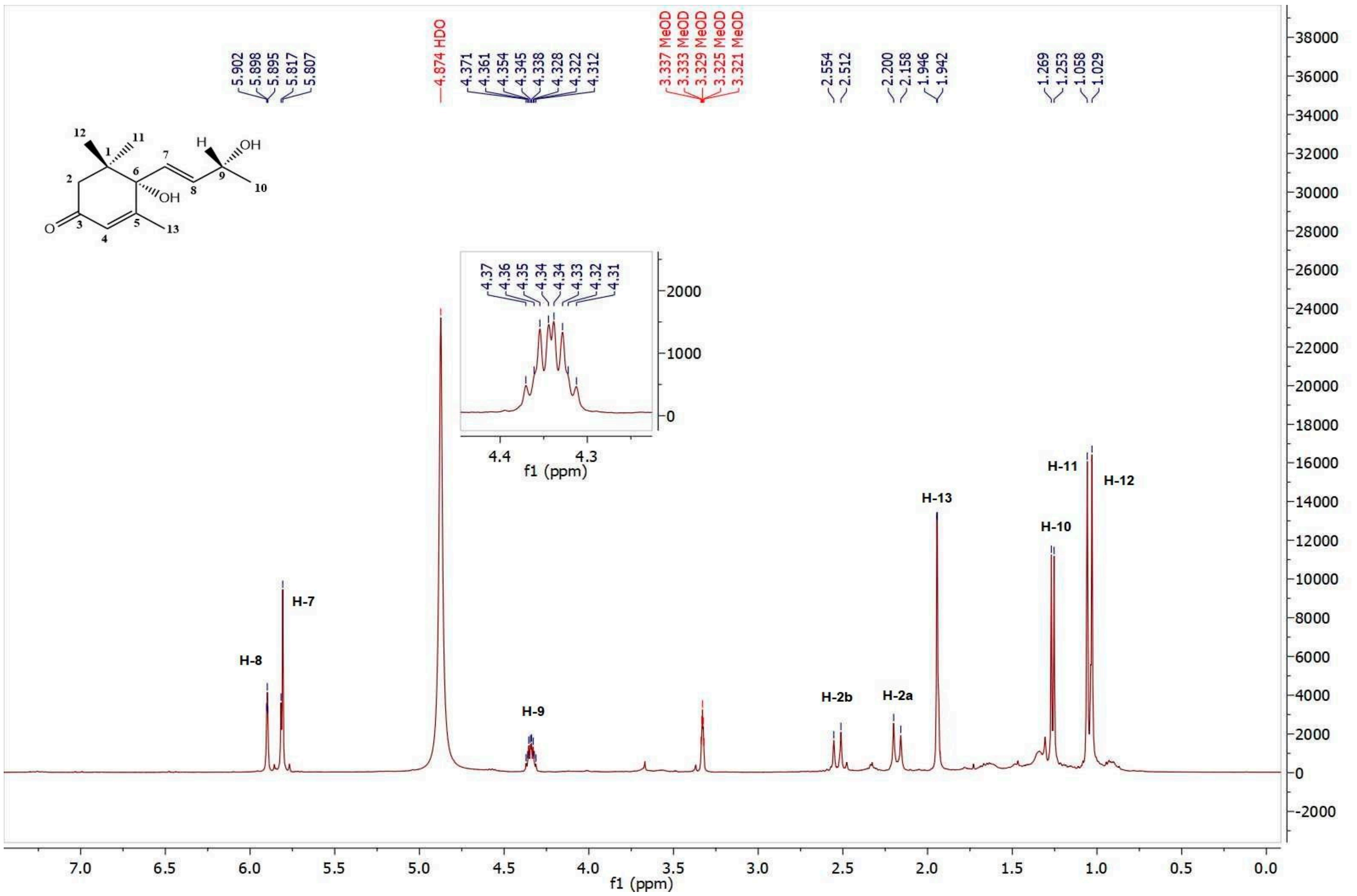

Figure S16: ^{1}H NMR spectrum of compound **(8)** Vomifoliol**.**

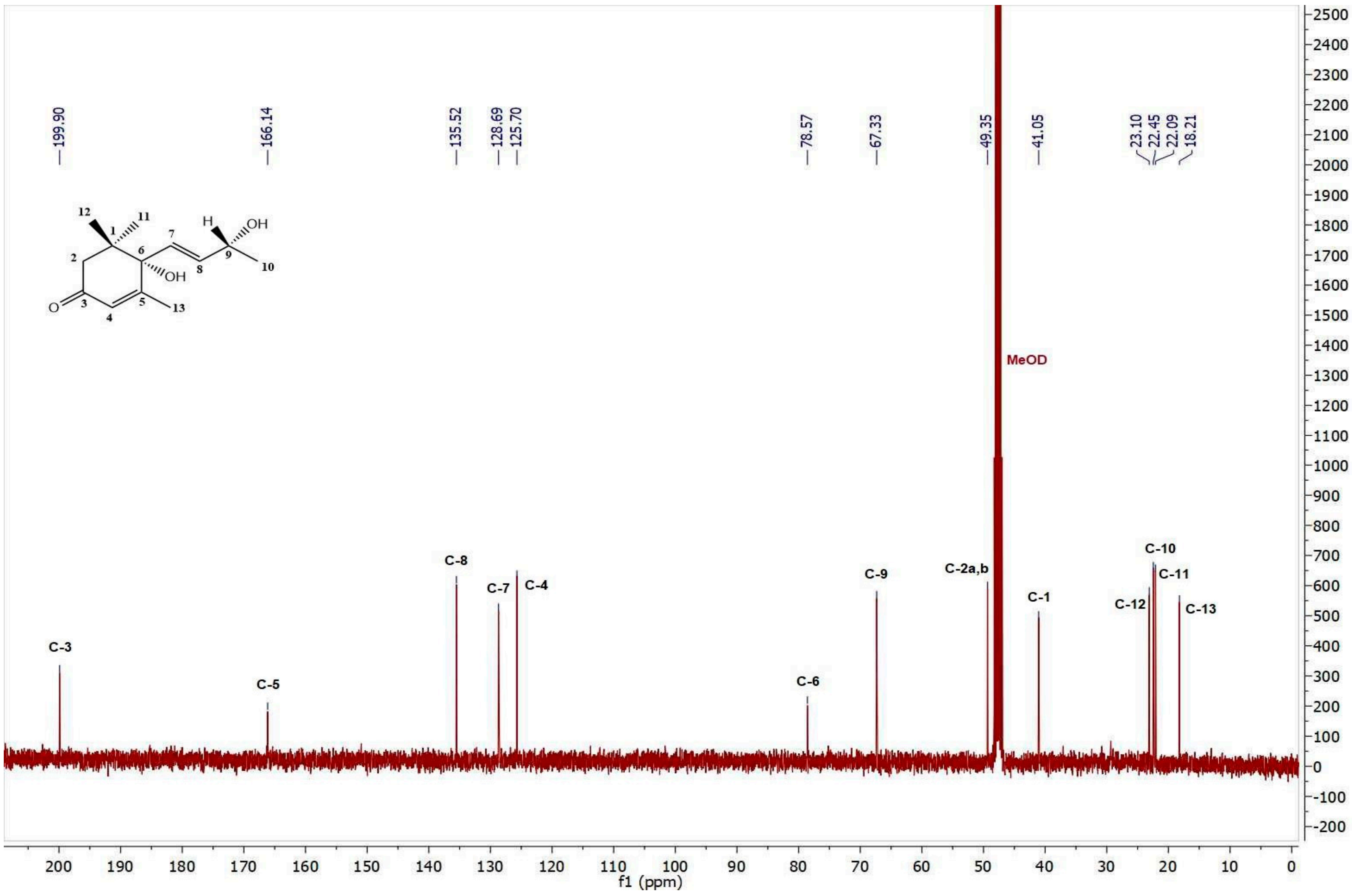

Figure S17: ^{13}C NMR spectrum of compound **(8)** Vomifoliol.

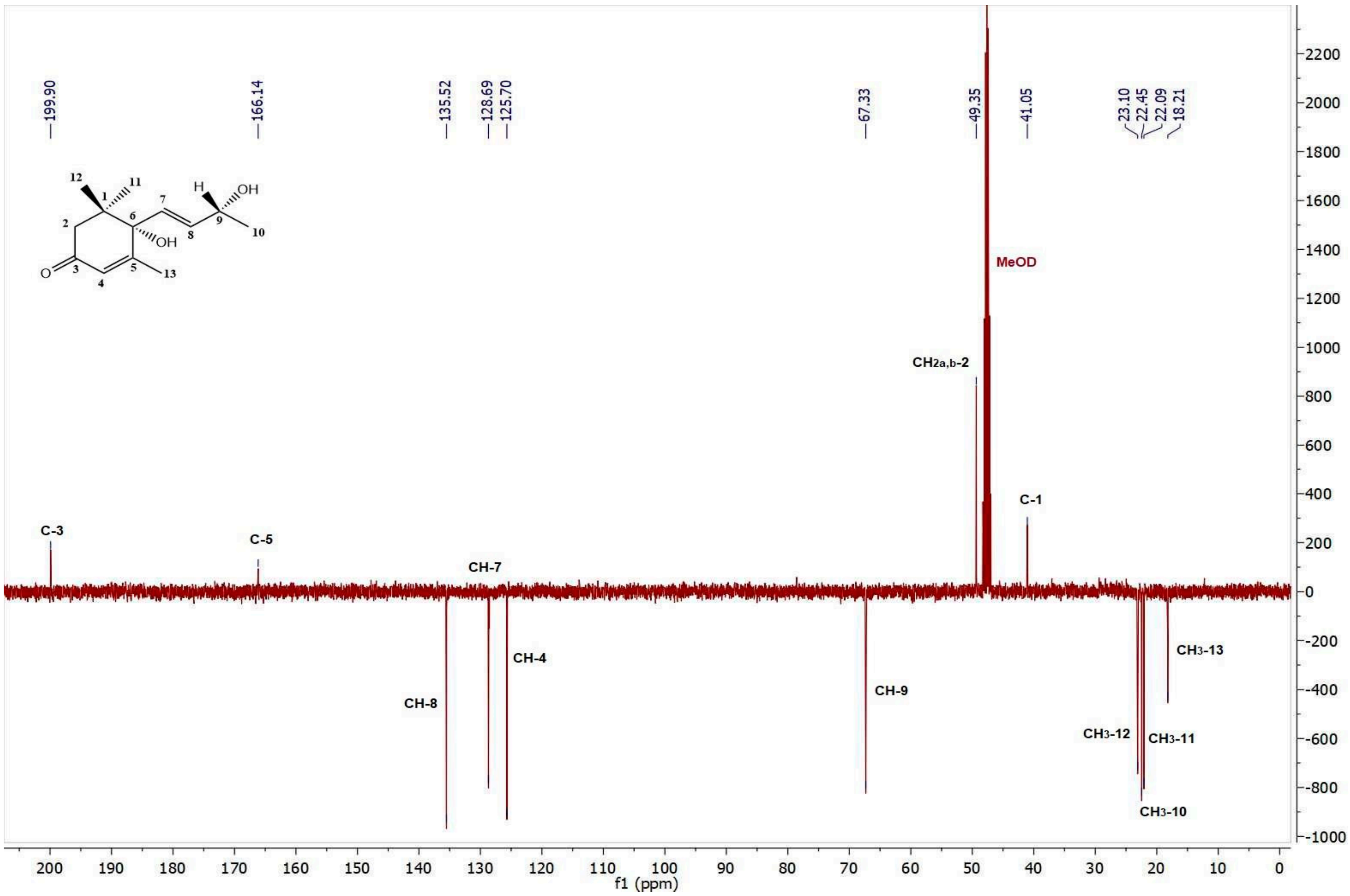

Figure S18: ^{13}C APT NMR spectrum of compound **(8)** Vomifoliol.

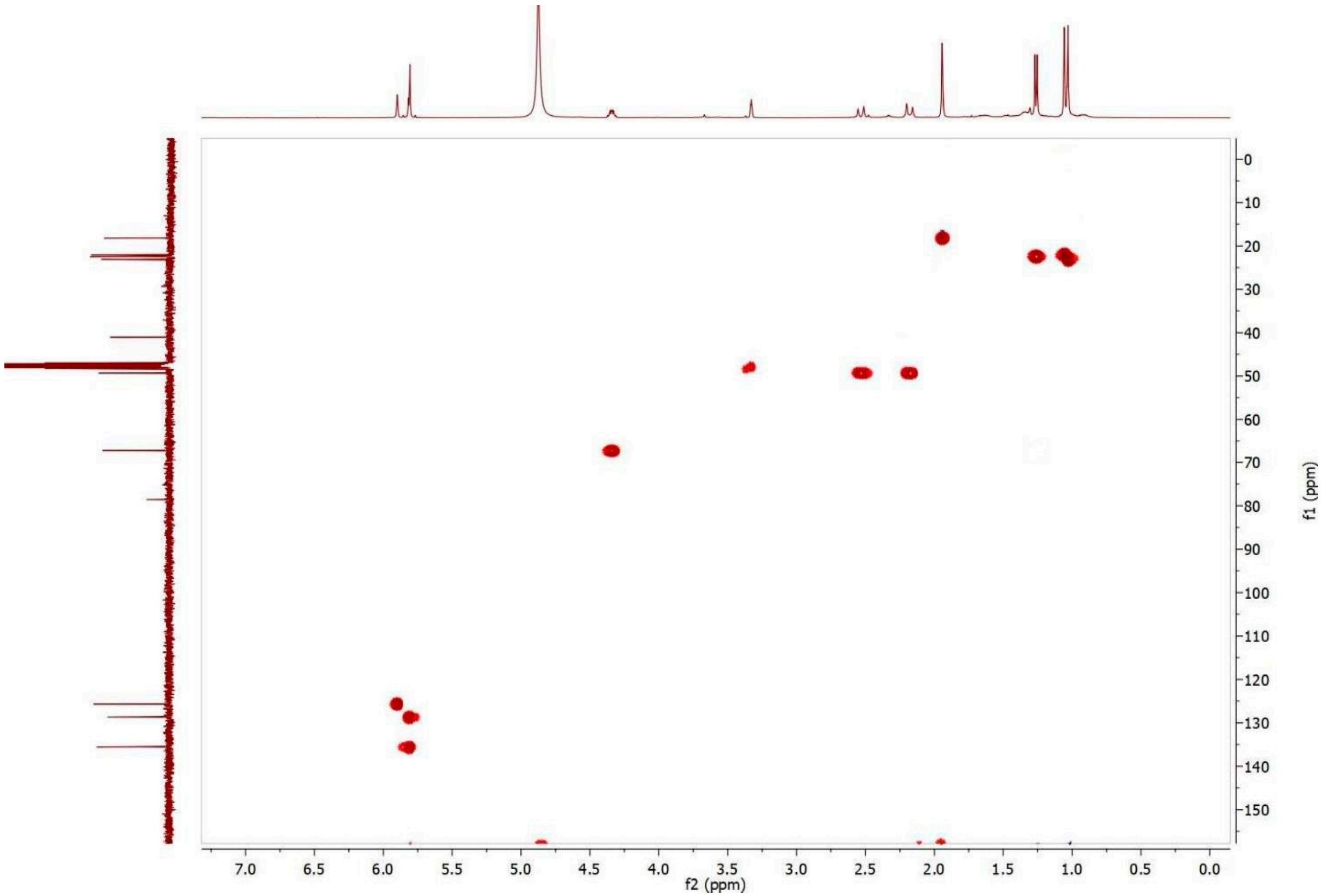

Figure S19: HSBC NMR spectrum of compound **(8)** Vomifoliol.

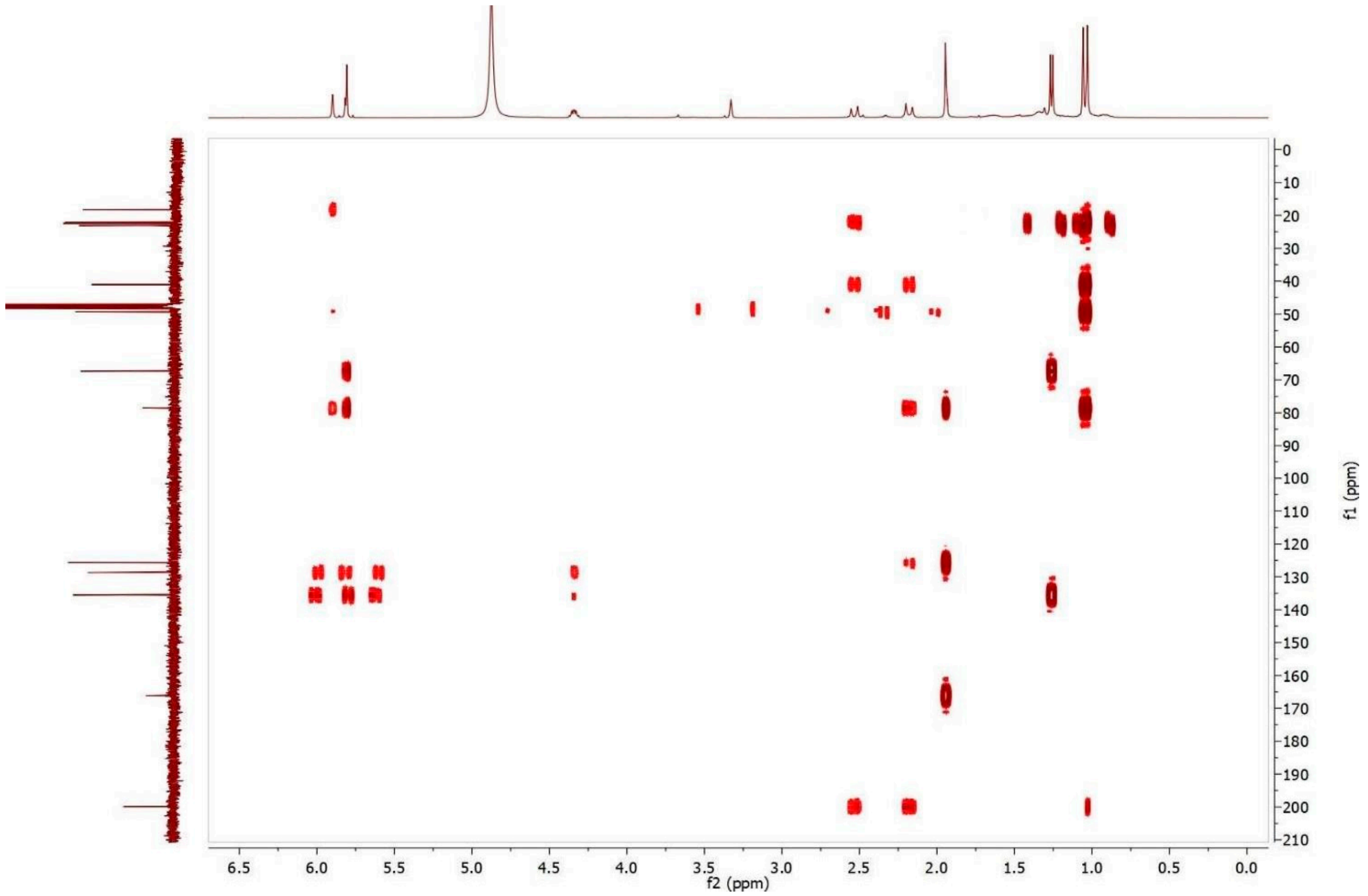

Figure S20: HMBC NMR spectrum of compound **(8)** Vomifoliol.

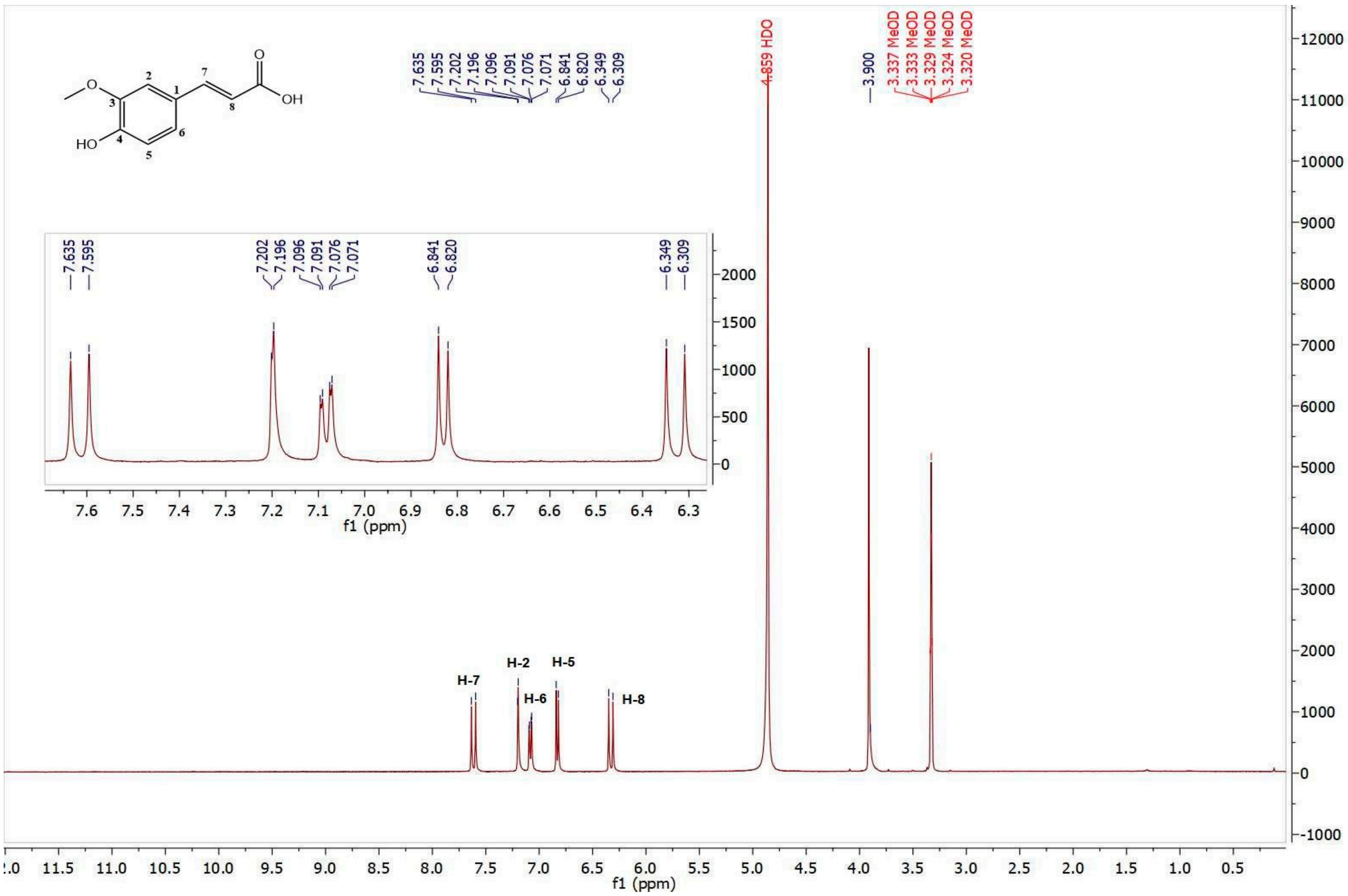

Figure S21: ^{1}H NMR spectrum of compound **(9)** Ferulic acid.

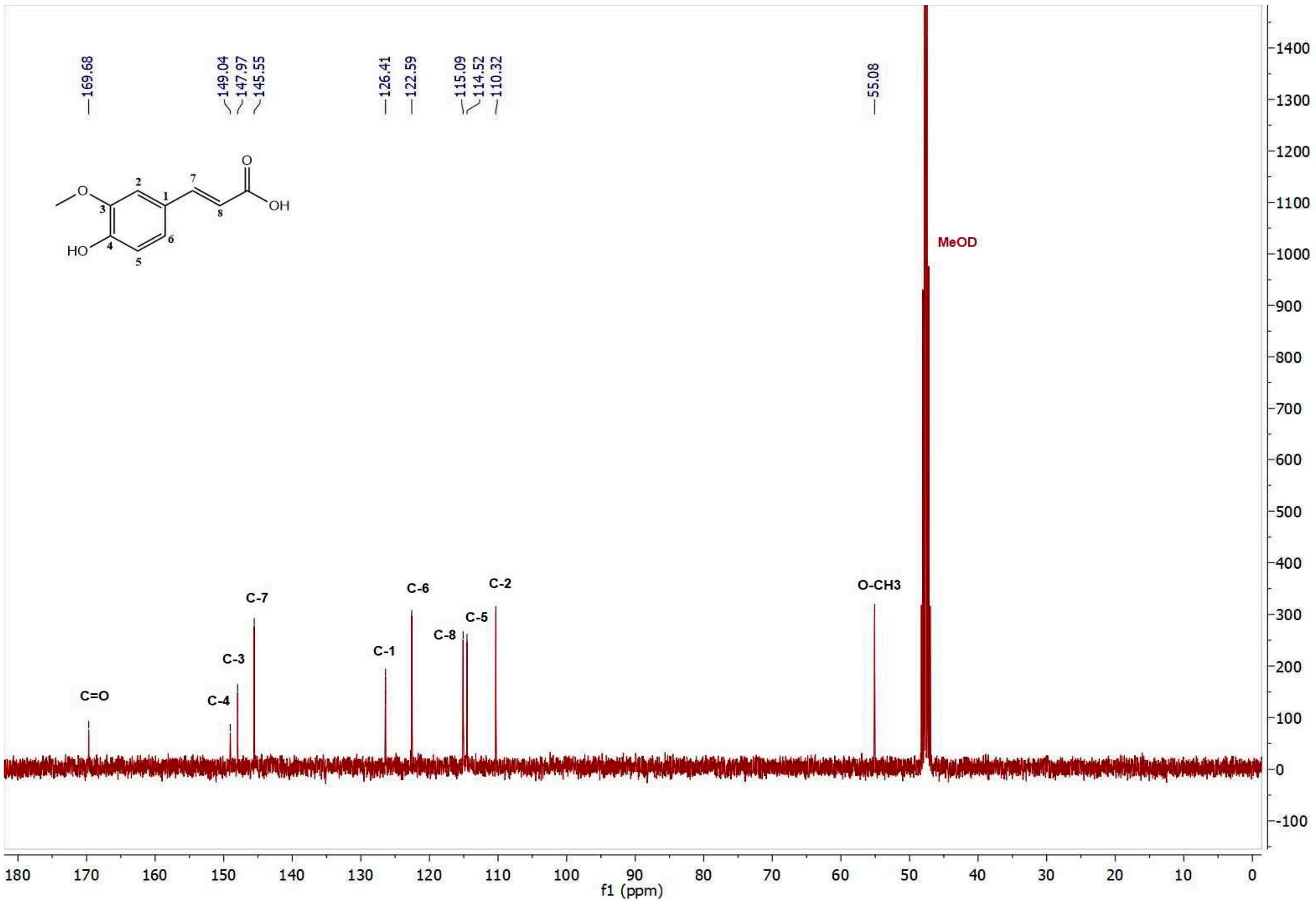

Figure S22: ^{13}C NMR spectrum of compound **(9)** Ferulic acid.

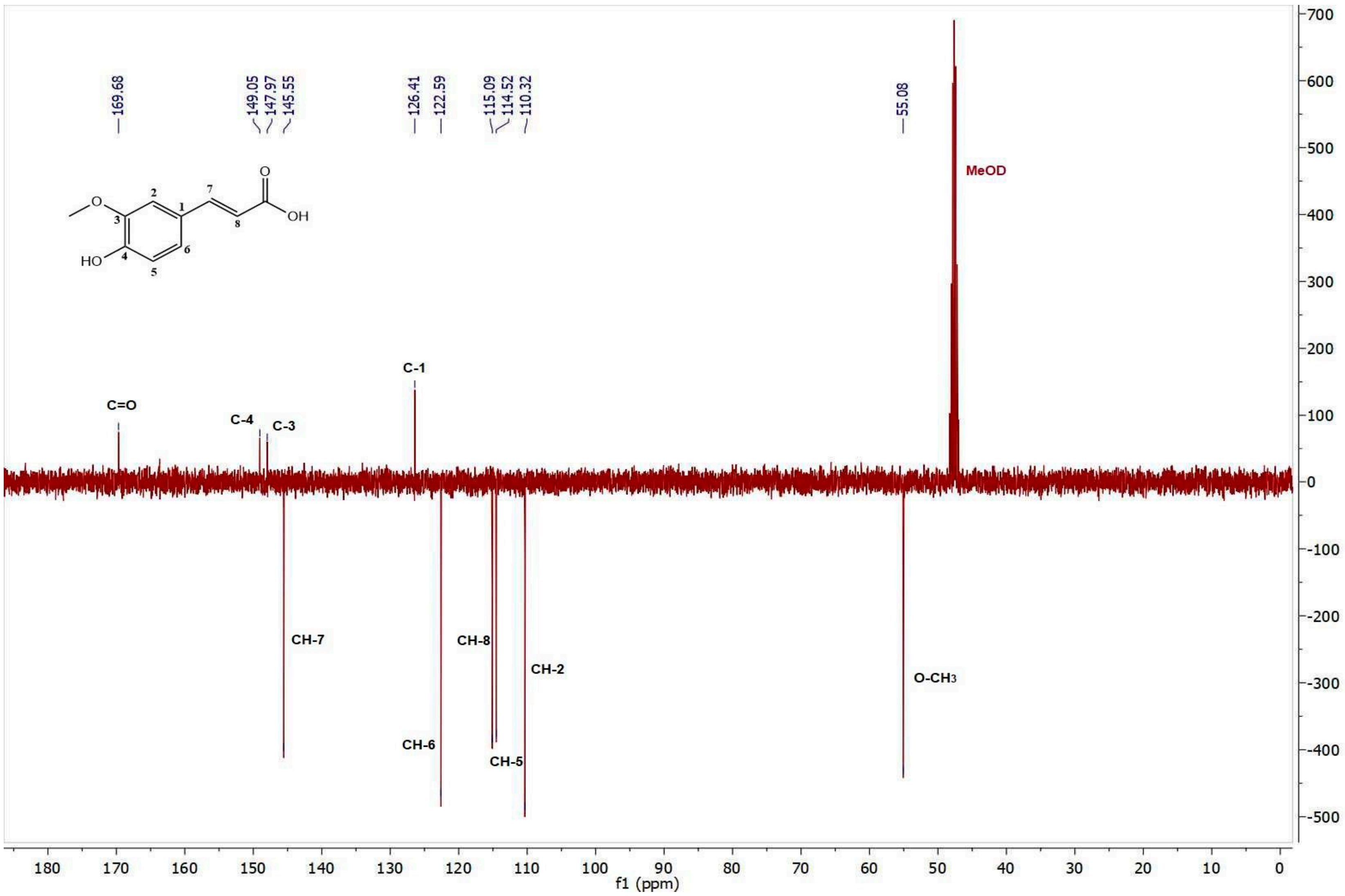

Figure S23: ^{13}C NMR APT spectrum of compound **(9)** Ferulic acid.

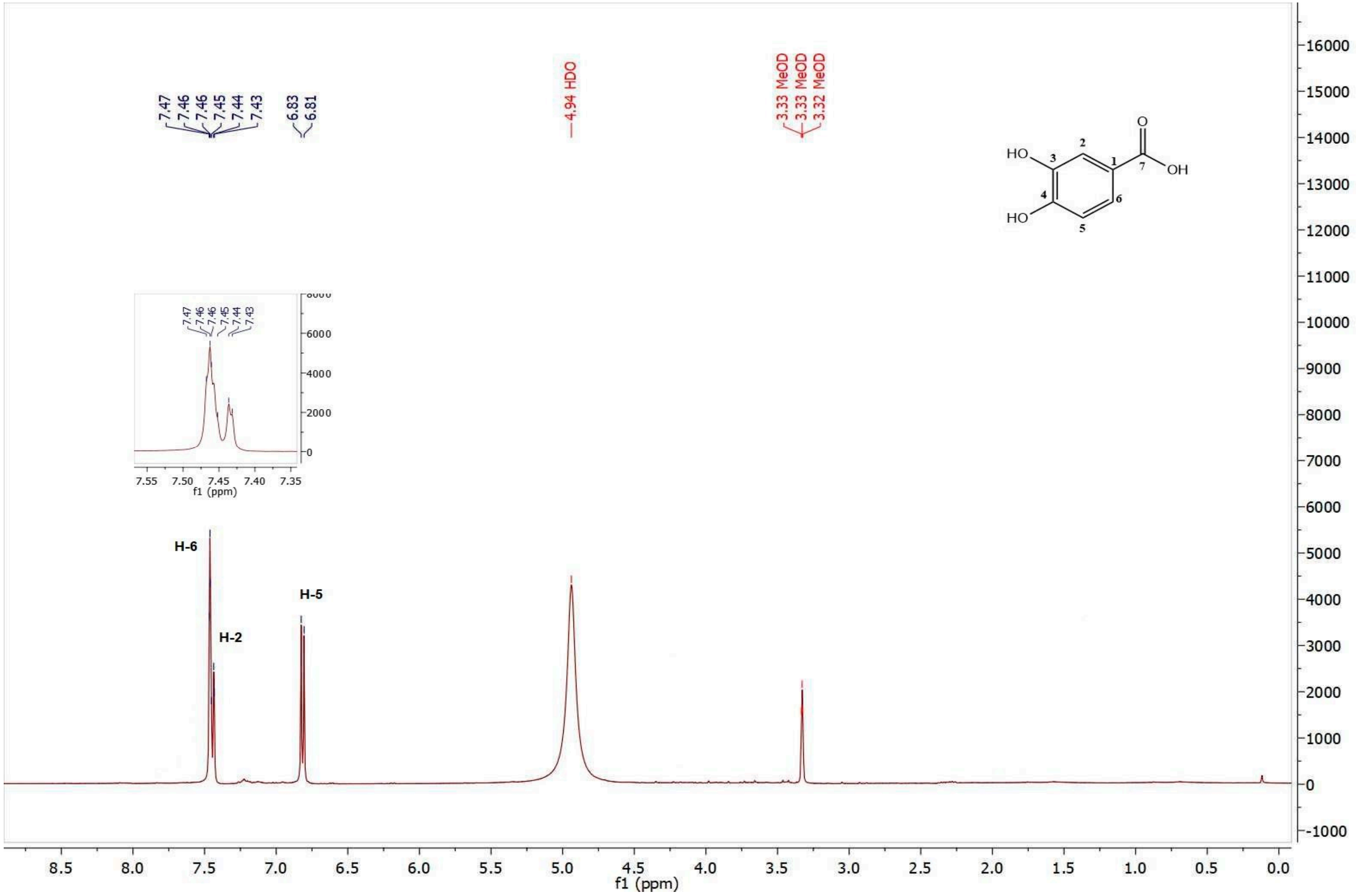

Figure S24: ^{1}H NMR spectrum of compound **(10)** Protocatechuic acid.

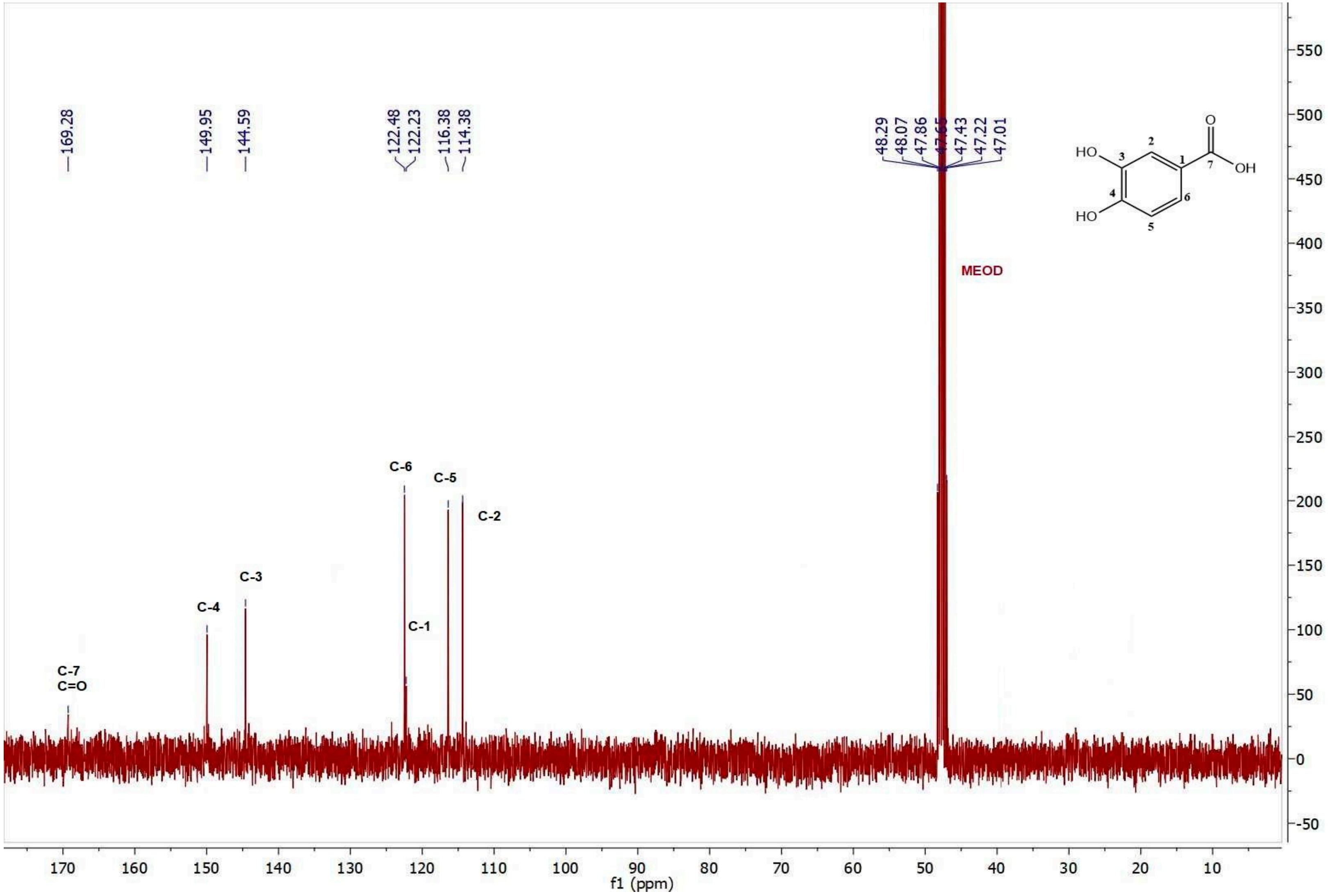

Figure S25: ^{13}C NMR spectrum of compound **(10)** Protocatechuic acid.

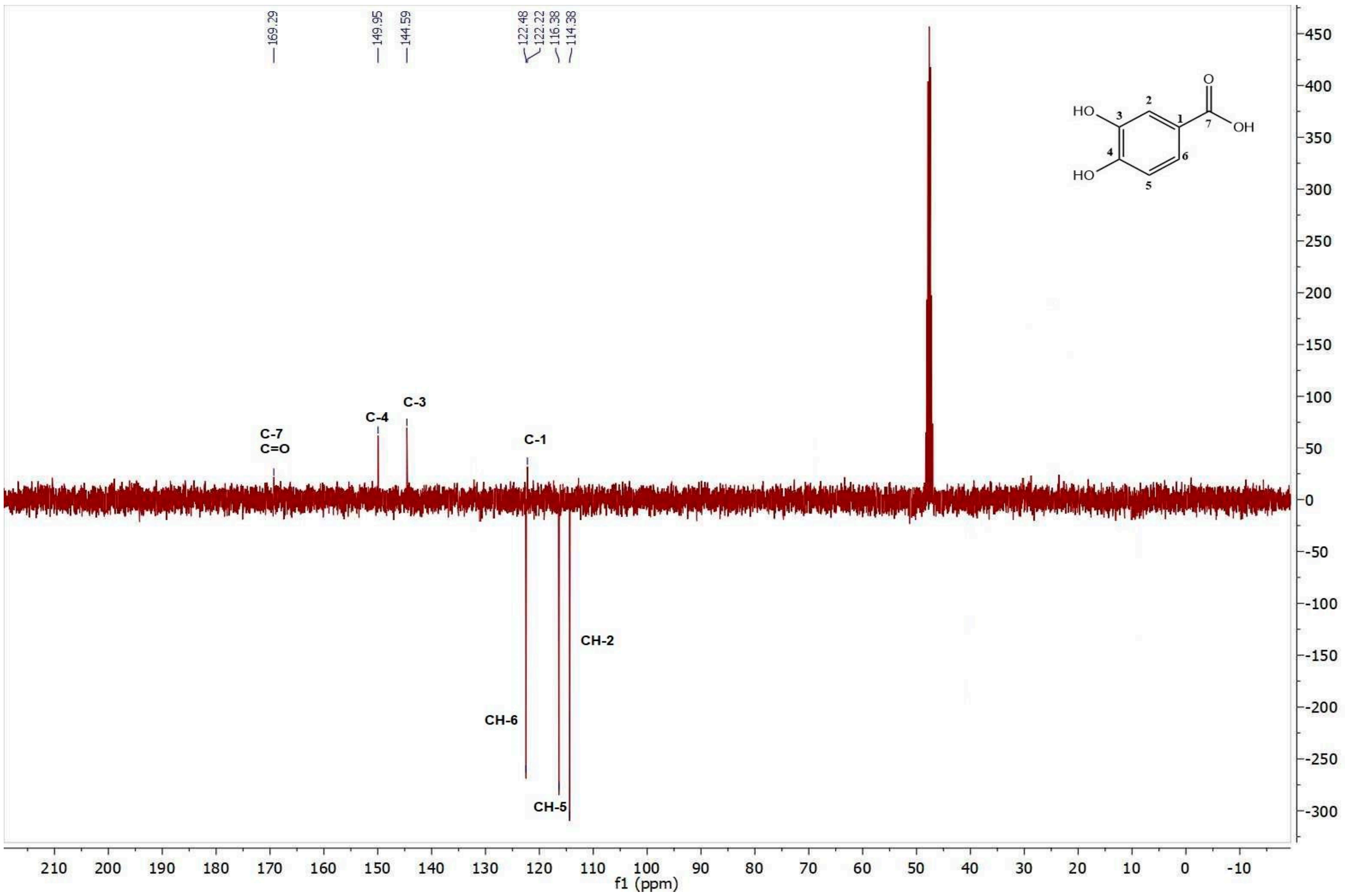

Figure S26: ^{13}C NMR APT spectrum of compound **(10)** Protocatechuic acid.

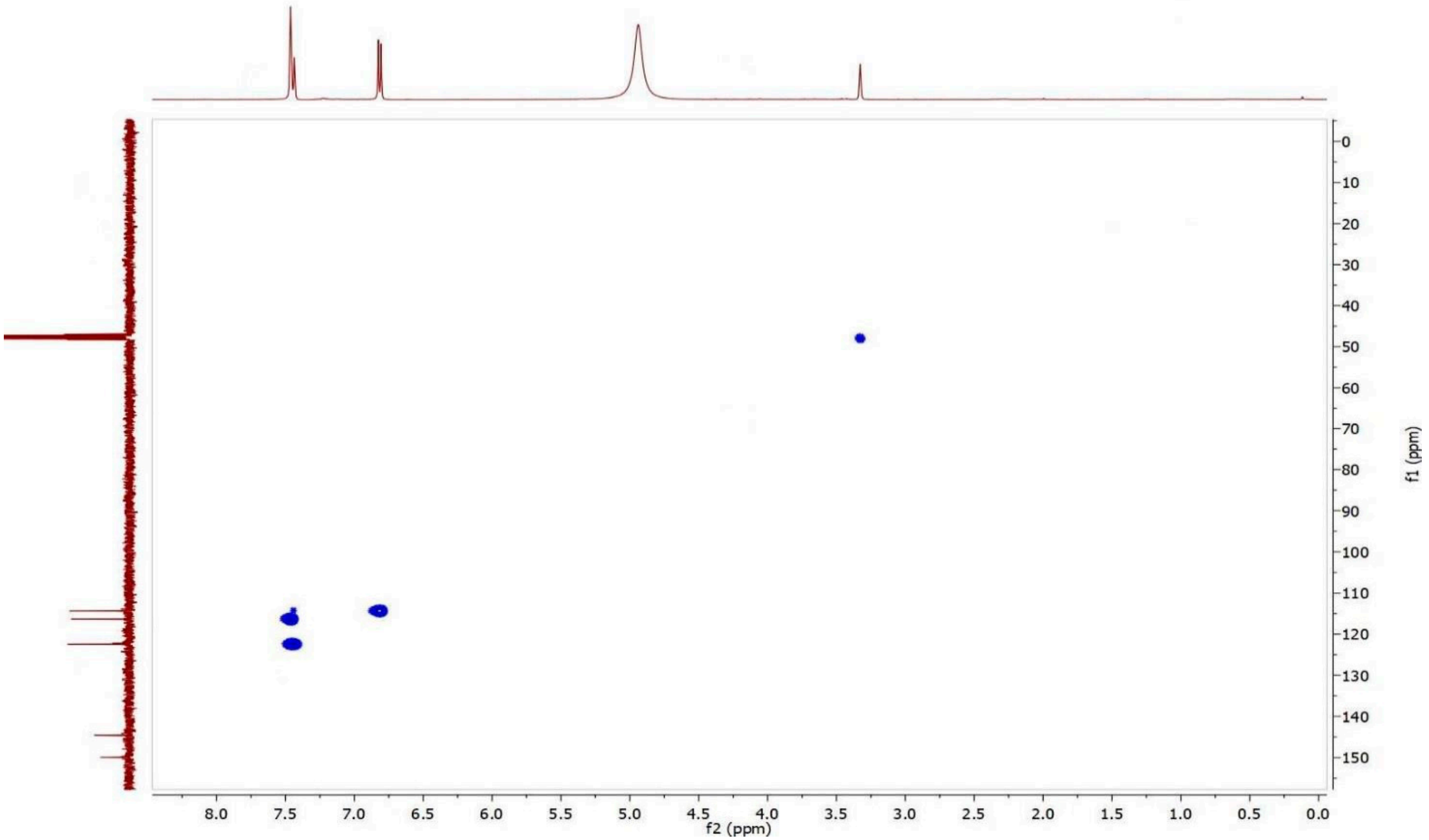

Figure S27: HSBC NMR spectrum of compound **(10)** Protocatechuic acid.

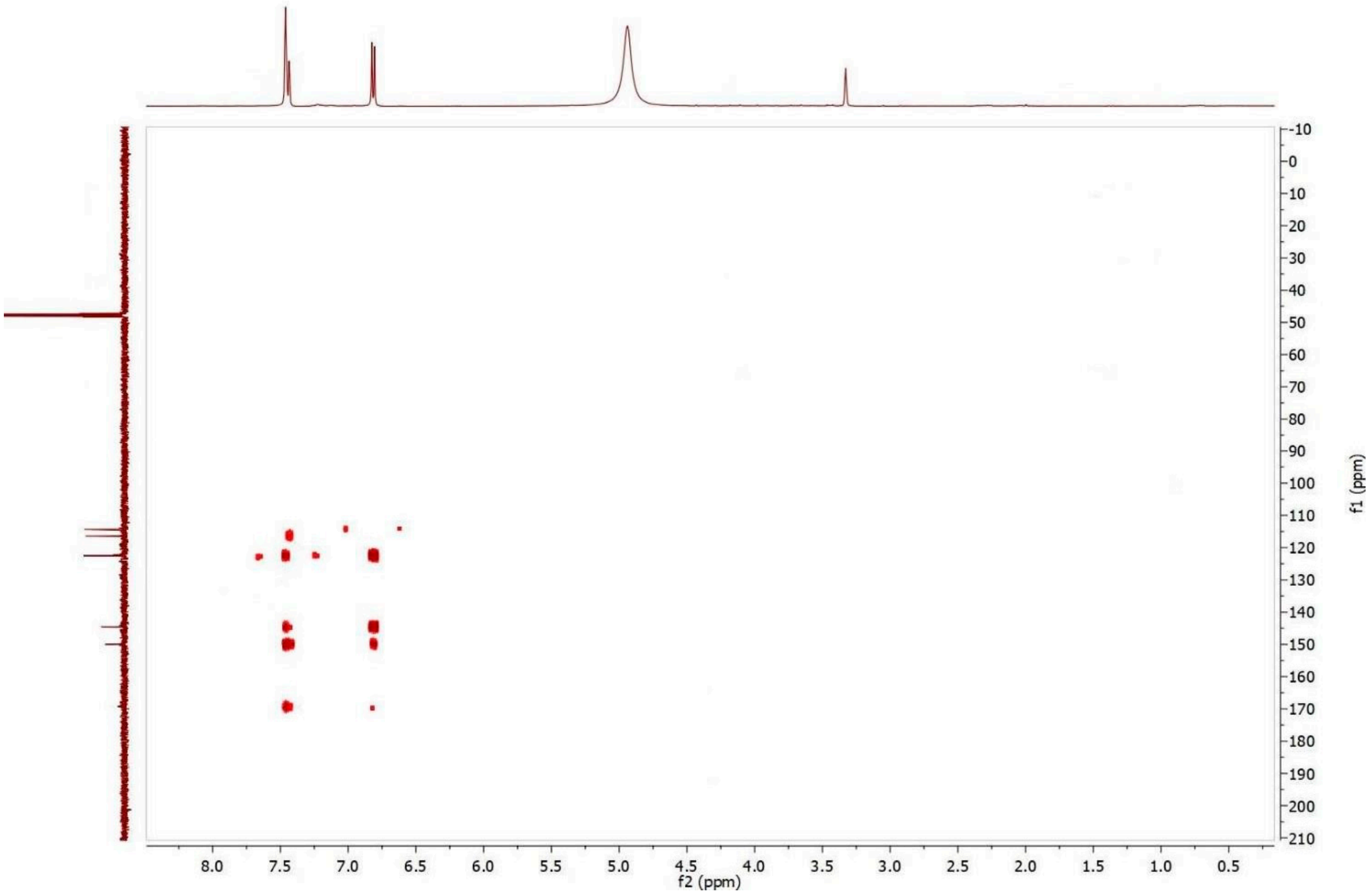

Figure S28: HMBC NMR spectrum of compound **(10)** Protocatechuic acid.

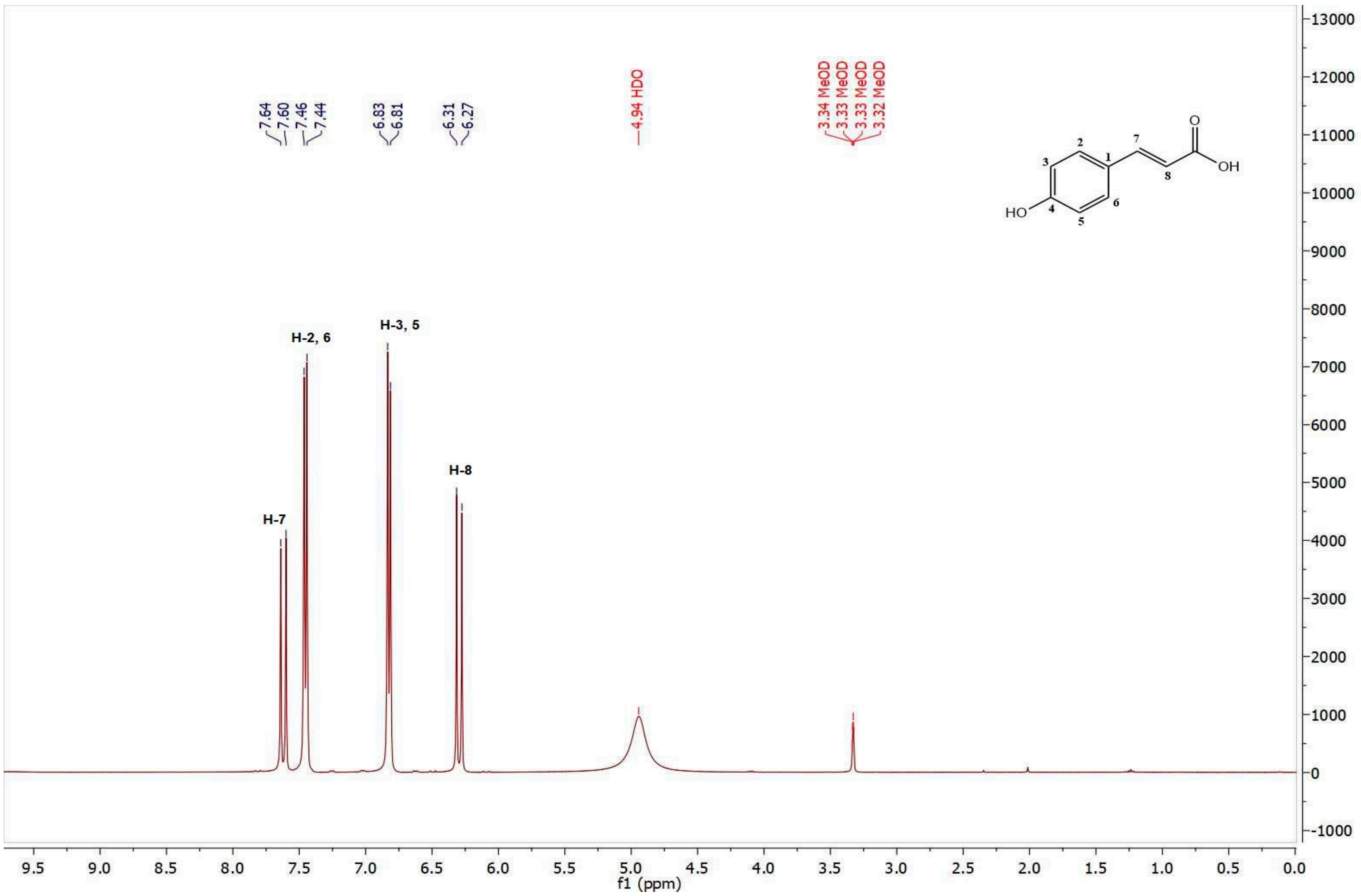

Figure S29: ^{1}H NMR spectrum of compound **(12)** *p*- Coumaric acid.

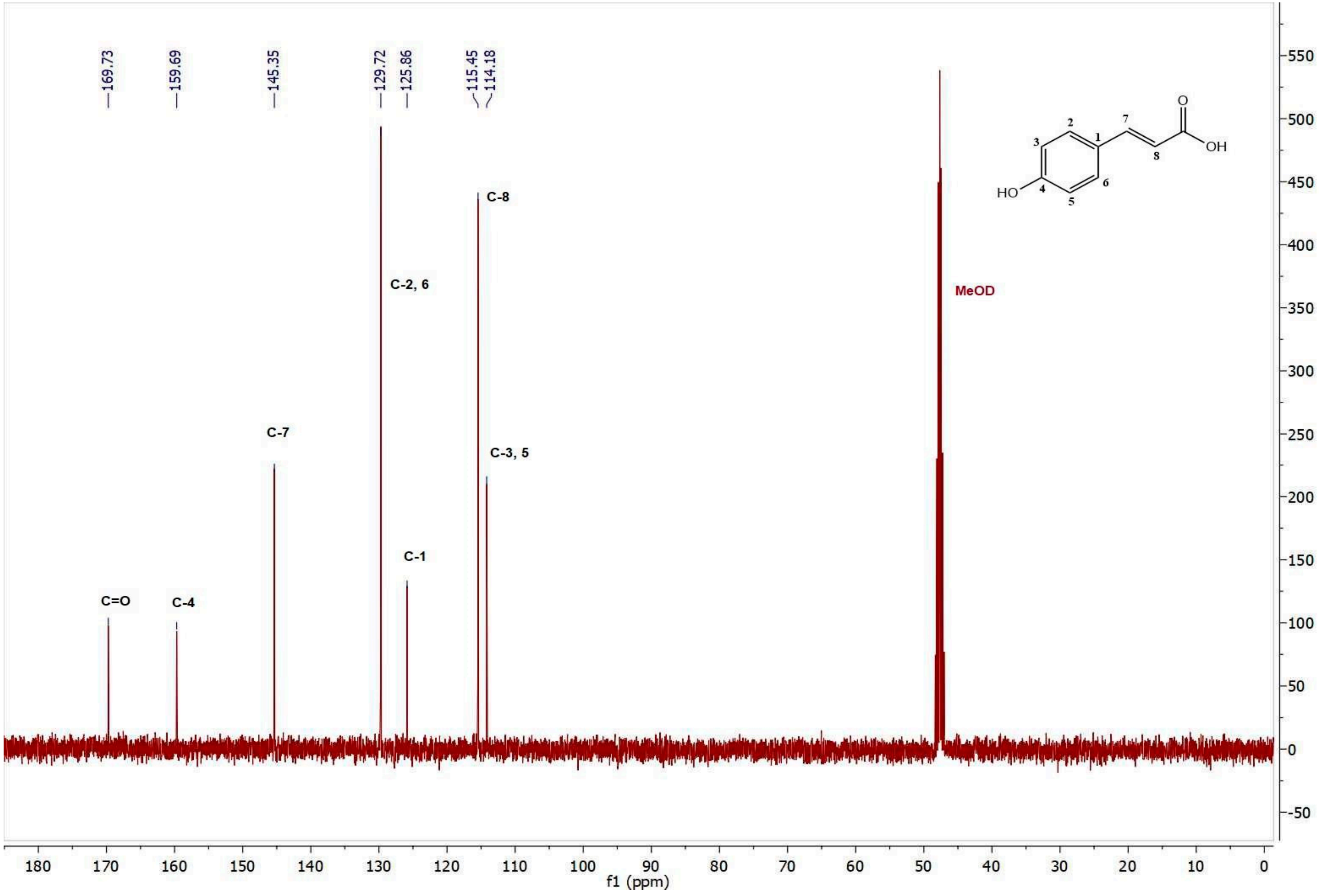

Figure S30: ^{13}C NMR spectrum of compound **(12)** *p*- Coumaric acid.

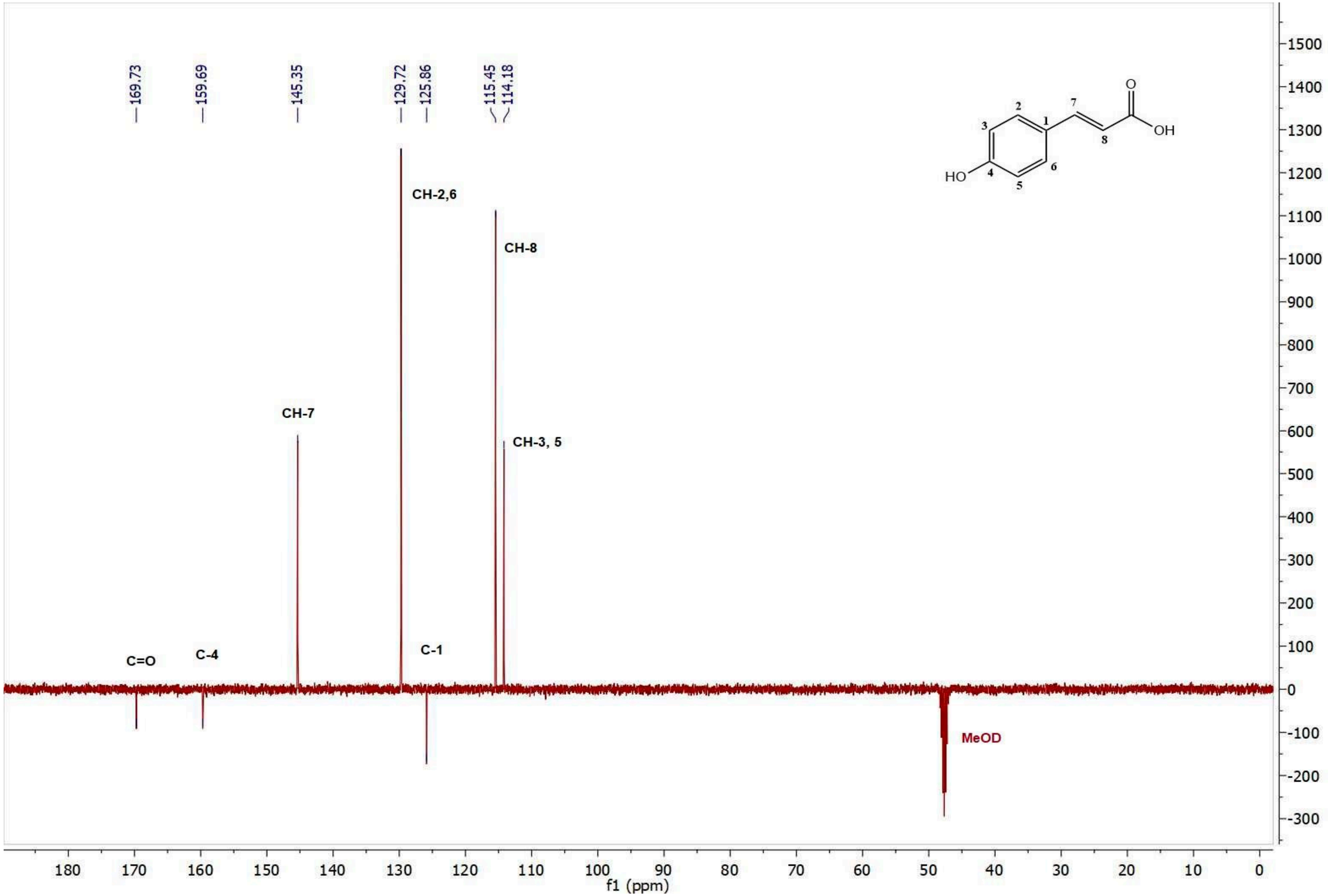

Figure S31: ^{13}C APT NMR spectrum of compound **(12)** *p*- Coumaric acid.
